# Impact of Long‐Term Drainage on Carbon Fluxes in the High‐Latitude Permafrost Region

**DOI:** 10.1111/gcb.70346

**Published:** 2025-07-15

**Authors:** Abdullah Bolek, Mark Schlutow, Theresia Yazbeck, Nathalie Triches, Martin Heimann, Mathias Göckede

**Affiliations:** ^1^ Max Planck Institute for Biogeochemistry, Biogeochemical Signals Jena Germany; ^2^ Institute for Atmospheric and Earth System Research University of Helsinki Helsinki Finland

**Keywords:** Arctic permafrost, CO_2_ and CH_4_ fluxes, drying or wetting, Eddy‐covariance, global climate change, high latitude ecosystems, permafrost disturbance

## Abstract

With Arctic amplification, hydrological conditions in Arctic permafrost regions are expected to change substantially, which can have a strong impact on carbon budgets. To date, detailed mechanisms remain highly uncertain due to the lack of continuous observational data. Considering the large carbon storage in these regions, understanding these processes becomes crucial for estimating the future trajectory of global climate change. This study presents findings from 8 years of continuous eddy‐covariance measurements of carbon dioxide (${\mathrm{CO}}_{2}$) and methane (${\mathrm{CH}}_{4}$) fluxes over a wet tussock tundra ecosystem near Chersky in Northeast Siberia, comparing data between a site affected by a long‐term drainage disturbance and an undisturbed control site. We observed a significant increasing trend in roughness lengths at both sites, indicating denser and/or taller vegetation; however, the increase at the drained site was more pronounced, highlighting the dominant impact of drainage on vegetation structure. These trends in aboveground biomass contributed to differences in gross primary production (GPP) between the two sites increasing over the years, continuously reducing the negative effect of the drainage disturbance on the sink strength for ${\mathrm{CO}}_{2}$. In addition, carbon turnover rates at the drained site were enhanced, with ecosystem respiration and GPP consistently higher compared to the control site. Because of the artificially lower water table depth (WTD), ${\mathrm{CH}}_{4}$ emissions at the drained site were almost halved. Furthermore, drainage altered the ecosystem's response to environmental controls. Compared to the control site, the drained site became slightly more sensitive to the global radiation ($R_{g}$), resulting in higher ${\mathrm{CO}}_{2}$ uptake under the same levels of $R_{g}$. Meanwhile, ${\mathrm{CH}}_{4}$ emissions at the drained site showed a higher correlation with deep soil temperatures. Overall, our findings from this WTD manipulation experiment show that changing hydrological conditions will significantly impact the Arctic ecosystem characteristics, carbon budgets, and ecosystem's response to environmental changes.

## Graphical abstract

Increasing air temperatures and changes in snow regimes in Arctic permafrost regions affect hydrological conditions, impacting ecosystems, vegetation structures, and carbon budgets. This study investigates the effects of hydrological changes on ecosystem dynamics by artificially draining a field site with a 200‐m diameter circular ditch, creating a dry ecosystem. A wet, undisturbed ecosystem located approximately 600 m away served as a control site. We observed significant differences in vegetation and methane fluxes between these sites. Additionally, ecosystem respiration was consistently higher at the dry site compared to the wet control site.

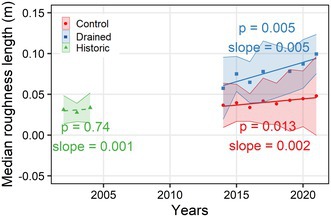

## 1 Introduction

Increasing air temperatures and changing snow regimes destabilize permafrost in northern high latitudes (Biskaborn et al. 2019), posing a risk of further amplifying global warming by exposing the vast amount of carbon stored within the Arctic permafrost regions to degradation (Kittler et al. 2016; Berner et al. 2020). Although there is some uncertainty in the literature regarding the land surface coverage of permafrost in the Northern Hemisphere (Obu 2021; Biskaborn et al. 2019; Boike et al. 2018), ranging between 15% and 25%, the estimated amount of carbon stored within this region is about 33% of the total global soil carbon pool, which is at least twice the amount found in atmosphere (Schuur et al. 2022; Rodenhizer et al. 2023). This carbon might be released to the atmosphere in form of greenhouse gases (GHG) such as carbon dioxide (${\mathrm{CO}}_{2}$) or methane (${\mathrm{CH}}_{4}$), which can potentially intensify global warming. Increasing temperatures may lead to more permafrost thaw, triggering increased decomposition, respiration, and gross primary production (GPP) due to an extended growing season or enhanced availability of soil nutrients (Belshe et al. 2013; Kwon et al. 2016; Flanagan and Syed 2011). Higher air temperatures also change the vegetation community structure, for example, by promoting increased vegetation heights and/or densities, or a shift towards more shrub abundance in some ecosystems (Myers‐Smith et al. 2019; Berner et al. 2020; Göckede et al. 2019). However, it still remains highly uncertain what the net response of Arctic permafrost ecosystems to these changes will be, and how this will influence the permafrost carbon feedback (Schuur et al. 2015). This uncertainty can to a large degree be attributed to the scarcity of observational datasets from the Arctic permafrost region, particularly with respect to year‐round measurements.

Changing climate conditions hold the potential to modify permafrost ecosystems in multiple aspects that are often closely connected. As one example, ice wedges, that is, networks of massive belowground ice features that are typically observed in permafrost regions (Liljedahl et al. 2016), may start degrading due to increasing temperatures, leading to changes in the landscape that can substantially alter ecological and hydrological conditions in the permafrost regions (Jorgenson et al. 2006; Kittler et al. 2016). The degradation of the ice wedges can lead to a small‐scale patchwork of either wetter or drier conditions, linked to the lateral transportation of the water. With water table depth (WTD) being considered as one of the main drivers of carbon budgets in Arctic permafrost regions (Zona et al. 2012; Natali et al. 2015), ice wedge degradation may affect vegetation and microbial dynamics, soil thermal regimes, snow cover dynamics, and the radiation budget (Göckede et al. 2016). Moreover, while it was shown that Arctic warming will promote plant productivity, limitations in soil water availability following drainage may counteract this effect in affected regions (Zona et al. 2023). Higher soil water content might change soil temperatures and the depth of the active layer due to increased heat conductivity under wetter conditions (Zona et al. 2012; Subin et al. 2013). These alterations in the ecosystem can change the carbon budget over permafrost regions and potentially shift these high‐latitude ecosystems from a net sink to a source of ${\mathrm{CO}}_{2}$. In addition, soil moisture is known to be one of the major controlling parameters of ${\mathrm{CH}}_{4}$ emissions as it regulates the extent of anaerobic zones in the soil column, which are important for methanogenesis (Natali et al. 2015; Sturtevant et al. 2012). Therefore, the impact of potential shifts of the water table depth on the ecosystem needs to be investigated to elucidate future feedback between climate and carbon in the permafrost region.

To quantify the carbon budgets of these high‐latitude ecosystems, long‐term observations are required year‐round, including both the growing and non‐growing seasons. The non‐growing season was found to be a significant carbon source for greenhouse gases, which is mainly due to microbial decomposition that can persist even under sub‐zero temperatures (Oechel et al. 2014; Rößger et al. 2022; Natali et al. 2019). With increasing temperatures over the Arctic permafrost regions, this decomposition rate is expected to increase; hence, quantifying the carbon budget during the non‐growing season becomes essential. However, continuous observations of carbon fluxes from remote Arctic permafrost regions are challenging with respect to infrastructure and logistics. At the same time, operating continuous measurements in the harsh Arctic climate conditions is associated with special maintenance requirements, such as a regular de‐icing of instrumentation or high power demand for heating devices.

In this study, we present findings from a long‐term WTD manipulation experiment in Northeast Siberia. We investigate the impact of drainage on the tundra ecosystem mainly based on year‐round datasets for the period 2013–2021 from two identically instrumented eddy‐covariance towers, one within an area affected by artificial drainage and one within an undisturbed tundra section as control. In addition, observations from the period 2002 to 2005 are included to highlight long‐term trends. The main objective of this study is to assess the impact of long‐term drainage on the annual budgets of ${\mathrm{CO}}_{2}$ and ${\mathrm{CH}}_{4}$, including the contributions of different seasons. Moreover, our study investigates trends and interannual variability in observed carbon fluxes over a period of 8 years, and how these correlate with environmental parameters such as, for example, air and soil temperature. This almost decade‐long observational data provides novel insights into the impact of drainage on tundra ecosystem characteristics and carbon cycle processes, and will thus be valuable for projecting the future of the Arctic permafrost region under climate change.

## 2 Methodology

### 2.1 Site Specifications and Climate Variables

In situ observations using eddy‐covariance towers were conducted at the Ambolikha research site (Göckede et al. 2016), located on a floodplain of the Kolyma River in Northeast Siberia near the town of Chersky (central coordinates: 68°36′46″ N 161°21′27″ E). The site is underlain by continuous permafrost, and the vegetation community is classified as wet tussock tundra mainly dominated by tussock‐forming sedges *Carex appendiculata* and *Carex lugens* and cotton grasses *Eriophorum angustifolium* (Kwon et al. 2016). The field site is prone to minor flooding around the time of snow melt that typically occurs around late May or early June, causing the water level to reach up to 0.5 m above ground level on the site (Göckede et al. 2019, 2016; Kittler et al. 2016, 2017; Kwon et al. 2016).

The Ambolikha site setup includes two eddy‐covariance towers, one situated within a drainage ring system (drainage tower; 68°36′47″ N 161°20′29″ E, measurement height: 4.5 m a.g.l.), and a second tower placed in an undisturbed tundra section about 600 m away (control tower; 68°37′00″ N 161°21′03″ E, measurement height: 4.7 m a.g.l.). The artificial drainage system, built in 2004 (Merbold et al. 2009), consists of a circular ditch of about 200 m diameter and an average width of 2–3 m that is connected to the nearby Ambolikha river by an outlet channel of 300 m length. This system effectively lowers the water table after snow melt and spring flooding, and leads to predominantly dry top soil layers throughout the growing season in the drainage area. The tundra sections affected by the drainage experience a lowering of the water table by up to 30 cm (Göckede et al. 2016), creating conditions that strongly deviate from those in the predominantly inundated surfaces in the undisturbed control section. The seasonal dynamics of lateral flow patterns within both drainage and control areas, including short‐term dynamics linked to precipitation input, have been described in detail by Raab et al. (2024).

Both towers are equipped with the same flux instrumentation, including a 3D ultrasonic anemometer (USA1, METEK Meteorologische Messtechnik GmbH, Germany), open‐path (Li‐7500, Licor, USA, for ${\mathrm{CO}}_{2}$ and $H_{2}O$) and closed‐path gas analyzers (FGGA, Los Gatos Research, USA, for ${\mathrm{CO}}_{2}$, ${\mathrm{CH}}_{4}$, and $H_{2}O$). Ancillary meteorological sensors include probes for temperature and humidity (KPK 1/6‐ME‐H38, Mela, Germany), net radiation (CNR4, Kipp & Zonen, Netherlands), precipitation (tipping bucket rain gauge, Thies Clima, Germany), and atmospheric pressure (Gill Pressure Port, USA). Soil temperature probes (Pt‐100, Jumo GmbH, Germany) were installed at different depths (at surface, 4, 8, 16, 32, 64, and 128 cm below ground) to monitor belowground conditions, supplemented by moisture probes at 8 and 16 cm depth (ML‐2x, Delta‐t Messdienst GmbH, Germany). For further details on instrumental setup, please refer to Kittler et al. (2016).

**FIGURE 1:**
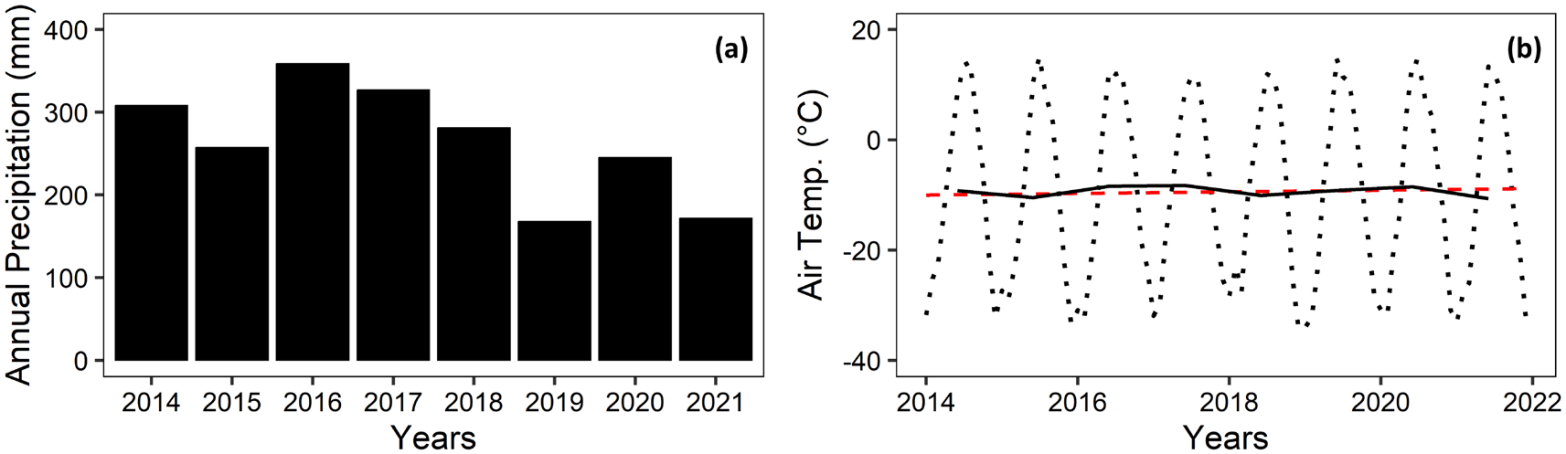
Climate variables observed at the Ambolikha field site: (a) annual cumulative precipitation (mm), (b) air temperatures (°C) over the study periods, with dotted lines showing monthly timesteps while solid line shows annual average air temperatures. The red dashed line represents the linear trend fitted to monthly averaged temperature data with a slope of 0.013°C ${\text{year}}^{-1}$.

The cumulative annual precipitation was reported as 197 mm between 1950 and 1999 (Kwon et al. 2016; Göckede et al. 2016), whereas from 2014 to 2021, it was measured as 251.6 mm (see Figure 1a for interannual variability). The years 2019–2021 were the driest period within the investigated timeframe, with particularly low values reported in 2019 and 2021. Due to biases in local precipitation measurements during the winter season, the shown precipitation data is a compilation of observations from the Ambolikha site and a meteorological station near Chersky (WMO ID = 25,123, 68°45′00″ N 161°16′59″ E; https://rp5.ru/Weather_archive_in_Cherskiy, last access: 29/11/2023). From the long‐term data record, the mean annual temperature for the Chersky weather station was reported as −11°C between 1961 and 2013 (Kwon et al. 2016; Göckede et al. 2016) whereas local measurements at the field site from 2014 to 2021 revealed a slightly warmer average temperature of −9.34°C. The difference between long‐term records and field site observations can at least partly be attributed to regional climate variability, since observation sites are situated about 15 km apart. The decadal temperature increase within the long‐term data record averages about 0.5°C, while between 2014 and 2021, our site measurements showed no clear trend in air temperatures. Figure 1b shows the long‐term monthly and annual air temperatures, with linear fitting to monthly averaged air temperatures indicated with a red dashed line. Although linear fitting to the monthly averaged temperatures shows a slope of 0.013°C ${\text{year}}^{-1}$, an opposite trend with a slope of −0.05°C ${\text{year}}^{-1}$ was observed in annual averaged air temperatures. During winter, monthly minima can fall below −30°C, whereas during the growing season (June–July–August), monthly maxima can be as high as 15°C. More details about the field site focusing on different aspects of the ecosystem and climate can be found in Corradi et al. (2005); Merbold et al. (2009); Kwon et al. (2016); Göckede et al. (2016); Kittler et al. (2016, 2017); and Raab et al. (2024).

### 2.2 Data Processing

The eddy‐covariance data were initially collected at a frequency of 20 Hz using the EDDYMEAS software package (Kolle and Rebmann 2010). Subsequently, the EddyPro software (www.licor.com/eddypro) was used to calculate the 30‐min averaged fluxes from the raw data. Double rotation was applied to wind data to align the mean wind speed along the stream‐wise direction. Various statistical tests, including spike removal (Mauder et al. 2013) and drop‐outs, were enabled to identify low quality data within the entire dataset. We adopted the scheme proposed by Foken et al. (2005) for general data quality assessment, with flags ranging from 1 (best) to 9 (worst), excluding data that received the lowest quality flag ratings of 8 and 9 from further analysis. We furthermore applied hard‐coded lower and upper thresholds to eliminate implausible data for the fluxes of ${\mathrm{CO}}_{2}$ (−15 and 5 $\text{μmol}\;m^{-2}\;s^{-1}$) and ${\mathrm{CH}}_{4}$ (−0.05 and 0.3 $\text{μmol}\;m^{-2}\;s^{-1}$) as well as for air temperature ($T_{a}$, −40°C and 60°C) (see also Kittler et al. (2017)).

Regarding flux data post‐processing, to address the density fluctuations in the open‐path data we applied the WPL correction (Webb et al. 1980). To account for the attenuation of eddy fluxes at high measurement frequencies, we applied the spectral correction method of Horst (1997) to the data. This choice follows the recommendation of Fratini and Mauder (2014) to make our results comparable with previous processing efforts by Kittler et al. (2016) who employed the TK3 software package (Mauder and Foken 2015) on the Ambolikha dataset. Nevertheless, the findings of this study partly deviate from the findings reported in Kittler et al. (2016, 2017) due to different pre‐ and post‐processing steps. Furthermore, we compensated for the self‐heating of the LI‐7500 by following the correction steps outlined in Kittler et al. (2017), which is a scaled version of the standard Burba correction (Burba et al. 2008). This heating of the instrument becomes particularly prominent in extremely cold environments (please refer to Kittler et al. (2017) for more details). For the final flux calculations, slowly measured (0.1 Hz) meteorological data including ambient pressure and temperature are required (see Kittler et al. (2016) for processing requirements). Estimates for flux uncertainties are provided by the EddyPro software by means of the random error method proposed by Finkelstein and Sims (2001).

In this study, we prioritize the closed‐path analyzer data over the open‐path data due to its higher overall accuracy. Nevertheless, the open‐path data were used to fill the gaps in closed‐path data whenever possible. As a preparation step, open‐path data during winter times were first subjected to a filter in order to reduce excessive noise. The site‐specific filter was based on the percentile range (i.e., 5% as a lower threshold, 80% as an upper threshold) of the weekly aggregated closed‐path ${\mathrm{CO}}_{2}$ fluxes between 2014 and 2021. Open‐path data that fell outside of this defined range were not used for gap filling.

**FIGURE 2:**
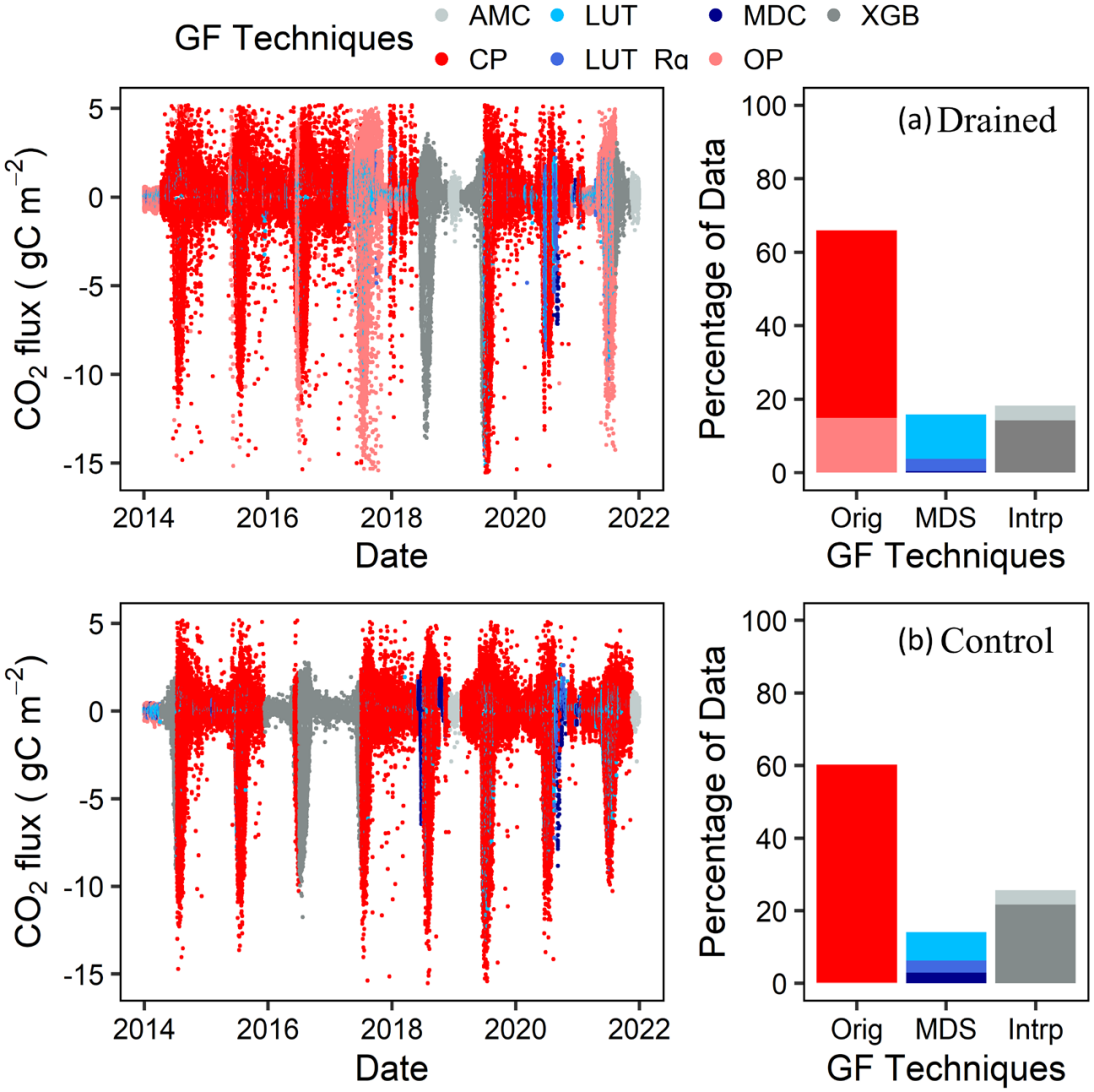
30‐min averaged ${\mathrm{CO}}_{2}$ fluxes with different gap filling (GF) techniques represented with different colors for (a) drained, and (b) control site. Here, AMC is annual mean course, XGB is gap filling technique uses the XGBoost machine learning model to predict the offset between two towers, LUT_Rg is look up table only use global radiation, OP is open path, CP is closed path, LUT is look up table, MDC is mean diurnal couse, Orig is original data coming either by CP or OP.

To produce continuous flux time series, we used a sequence of three different gap‐filling approaches: As the default gap filling for ${\mathrm{CO}}_{2}$ fluxes, we used the marginal distribution sampling (MDS) method (Reichstein et al. 2005) made available as part of the R package REddyProc (https://r‐forge.r‐project.org/projects/reddyproc/). Whenever MDS was not able to fill the gaps (i.e., for long gaps), they were filled by an algorithm that used data from the other tower, if available (i.e., for the drained tower, we used control tower data, and vice versa). Specifically, a machine‐learning algorithm (XGBoost model) was built using 10 different training and validation sets (90% for training and 10% for validation) of artificial gaps, which were then aggregated to ensure robustness. For each variable to be gap filled, a model was trained to predict the offset between the two towers using various model parameters and predictors (see Table S1 for more details and RMSE values). Finally, gaps were filled using predicted offsets and observations of the other tower. To prevent excessive outliers, predictions were constrained by the annual mean course (AMC), limited to AMC ($\pm 3\sigma_{\mathrm{AMC}}$), where ($\sigma_{\mathrm{AMC}}$) is the standard deviation of the AMC. If data from the other tower data were unavailable for longer gaps, for example, due to a power failure that affected both sites, the algorithm defaulted to using the AMC for each tower for gap filling. RMSE values for AMC gap filling were calculated using artificial gaps and are provided in Table S1. The resulting data coverage for ${\mathrm{CO}}_{2}$ fluxes from 2014 to 2022 is shown in Figure 2 for both drained and control sites, with colors reflecting the chosen gap filling (GF) techniques for each 30‐min averaged ${\mathrm{CO}}_{2}$ flux value. At both sites, at least 60% of the data were obtained as direct flux measurements from either closed‐path (CP) or open‐path (OP) gas analyzers (Orig). For ${\mathrm{CH}}_{4}$ fluxes, about 48.5% and 58% of the data were obtained from CP analyzer for drained and control site, respectively, with gaps filled using similar methods as for ${\mathrm{CO}}_{2}$ fluxes (see also Figure S1). As a final step in data processing, the gap filled ${\mathrm{CO}}_{2}$ fluxes were partitioned into GPP and Reco using a daytime partitioning (Lasslop et al. 2010) function provided within REddyProc. Although this approach generally predicted plausible results, occasional unphysical Reco values were observed during snow cover seasons. To avoid biases in the time series, these Reco signals were removed using a despiking algorithm (RFlux; Vitale et al. 2022). During late winter 2016 for the drained site and early winter 2014 for the control site, further processing was required as issues persisted. We replaced these periods with the average Reco from previous and following years for the same time period. These anomalies were most likely caused by the light‐response curve (LRC) being sensitive to the parameters employed for the fitting, such as, for example, VPD and uncertainty of ${\mathrm{CO}}_{2}$ fluxes (Wutzler et al. 2018). By creating artificial gaps, we calculated the RMSE linked to this processing step, which were 0.269 and 0.287 gC $m^{-2}{\mathrm{day}}^{-1}$ for the drained and the control site, respectively.

Apart from the recent dataset spanning from 2014 to 2022, we used a historical dataset collected in growing and fall seasons between 2002 and 2005 at the drained site to provide further insights into ecosystem dynamics (Corradi et al. 2005; Merbold et al. 2009; Kittler et al. 2016). As the drainage ditch was installed in late fall in 2004, the last year (2005) of this dataset reflects the disturbed ecosystem characteristics (i.e., drained) while the first 3 years represent the undisturbed ecosystem (i.e., control) characteristics. Note that the carbon fluxes of the historical dataset were processed using a different software (TK3, see Kittler et al. (2016) for details); therefore, comparisons need to be done cautiously, as biases may, to some extent, result from different flux processing software packages.

### 2.3 Statistics

For the analysis of seasonal trends and differences, the calendar year was divided into four different seasons: snow cover, spring, growing, and fall. For the snow cover season, the timing of snow cover onset and snow melt was determined using albedo data obtained from measurements of short‐ and long‐wave radiation at both sites. To avoid biases due to sensor view at each site, we used the latest date between the two towers for both snow cover onset and snow melt. As a plausibility check, we used phenocam images from the drained site to verify and correct the snow cover onset and melt dates. Such corrections, however, were only necessary for 2015 and 2019. In this context, the albedo threshold for identifying the establishment of a closed snow cover was set to 0.6, whereas for its disappearance at snow melt, we used a value of 0.3 (https://gml.noaa.gov/grad/snomelt.html, last access: 25/09/2024). To maintain continuity in the snow cover season, we determined the onset from the preceding calendar year, while its end is taken from the subsequent year. For instance, the 2014 snow cover season begins in late 2014 and ends in early 2015. The onset and the end of the growing season were defined as the times showing 50% of the maximum annual GPP. A recent study (Fang et al. 2023) investigated three different thresholds (10%, 25%, and 50%) to define the onset and end date of the growing season. Although the overall performance for 25% was found to be slightly better than for the 50% threshold in their study, here, we used 50% to avoid the influence of the annual flooding that takes place between late May and early June on the assignment of season start and end dates, with the aim to avoid uncertainties in the quantification of carbon fluxes during the early growing season. Spring and fall seasons were defined as the transition periods between snow melt and the beginning of the growing season (spring) or the end of the growing season and the onset of a closed snow cover (fall).

The observed differences between the drained and the control site were subjected to statistical tests using a two‐sample *t*‐test by bootstrapping the data with 10,000 replicates. To account for the relatively small sample size, we additionally conducted a Wilcoxon rank sum test, a non‐parametric test that does not rely on the assumption of normal distribution. Statistically significant differences between two sites are only reported when both tests indicate statistically significant results (from *t*‐test $p_{t}$ < 0.05 and from Wilcoxon test $p_{w}$ < 0.05). To investigate the relationships between the environmental controls and the carbon cycle processes, we first used partial correlation and random forest regression to identify important control variables for each carbon flux component. Subsequently, we applied both a simple linear regression (Natali et al. 2015; Kittler et al. 2017; Liu et al. 2020) and a non‐linear regression. Output from the latter was taken over whenever a better relationship than based on linear regression was observed. For this purpose, carbon fluxes and environmental controls were aggregated to monthly averages. During seasonal transitions, we imposed a minimum limit of 21 days as input per aggregated timestep. This limit was based on the minimum number of days available in the spring season, ensuring that each season‐year is represented by at least one data point. For each analysis, the correlation coefficient (r and τ for linear and non‐linear correlations, respectively) and corresponding (*p*) values were reported.

## 3 Results and Discussion

### 3.1 Trends in Site Characteristics

#### 3.1.1 Length and Onset of Seasons

**FIGURE 3:**
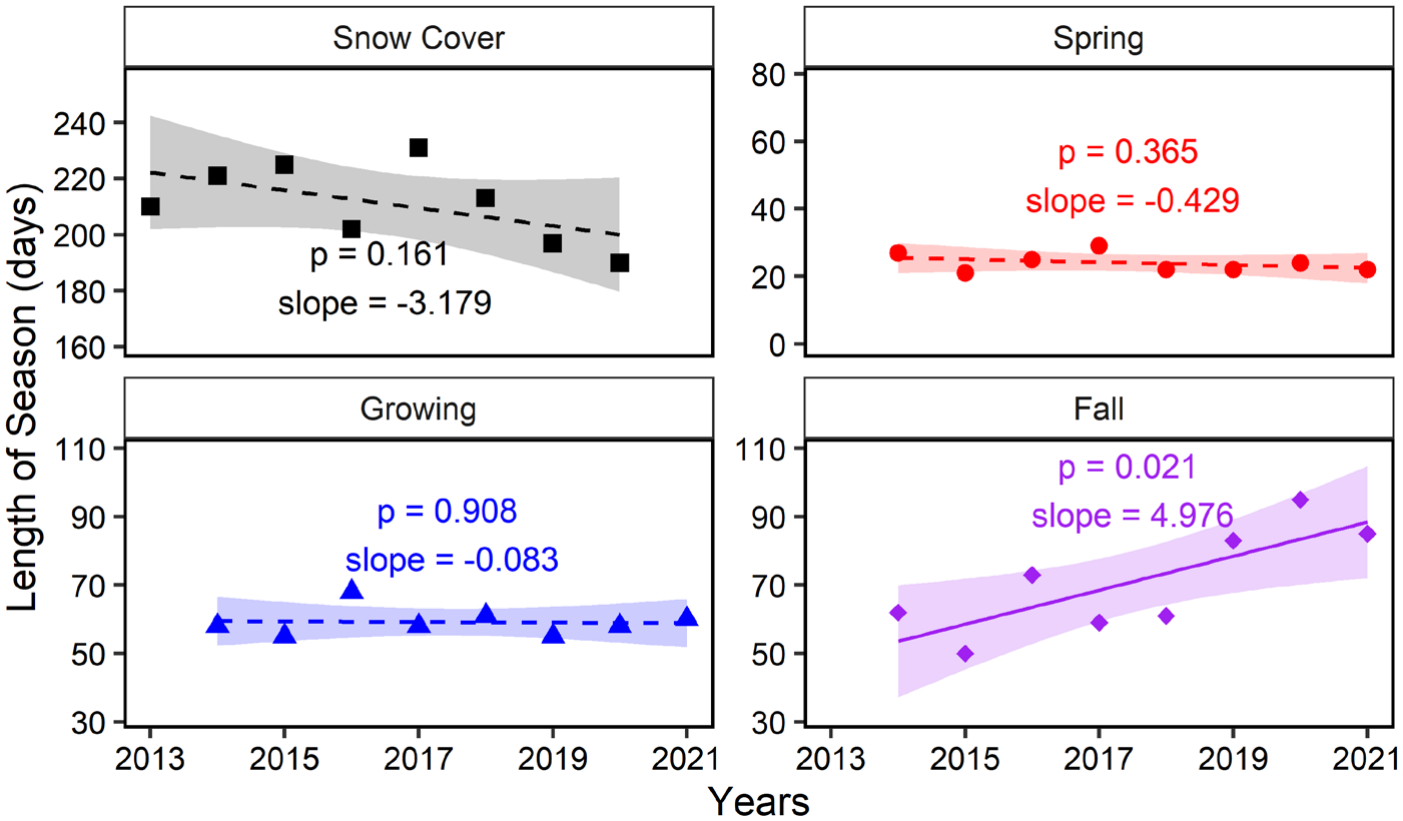
Changes in the length of the seasons over the years where each panels indicate different seasons. Here, each symbol represents the length of the corresponding season. For the snow cover season, the year indicates the start of the season, that is, the data point for 2013 indicates the length of the 2013/2014 season. The solid line denotes the significant trends (p<0.05), while dashed lines are non‐significant trends (p>0.05) for the fitted linear regressions over time. The shaded areas representing the 95% confidence interval while the slope values were provided for each trend with a unit of (day ${\text{year}}^{-1}$).

Figure 3 shows the length of each season obtained from observational data for the period 2014–2021 (except the snow cover season, which starts in 2013 and ends in 2020 due to our definition of the season). Only minor changes were found in the length of the growing and spring seasons over these 8 years, with the growing season remaining particularly stable at around 59 ± 4 days. The length of the snow cover and fall seasons, however, changed substantially over the same period. The snow cover duration at our field site decreased by about 23 days, with the average length of the snow cover season being 217 ± 10 days until 2019 (2013–2018), and 194 ± 5 days from 2019 onward (2019–2020). This decrease was compensated by an approximately equal increase (~27 days) in the length of the fall season, with the average length being 61 ± 8 days until 2019 (2014–2018), and 88 ± 6 days from 2019 onward (2019–2021).

The onset of each season, expressed as day of year (DOY), is provided in Table 1. Except for the snow cover season, all seasons tend towards starting slightly earlier over the years, about 7, 9, and 12 days earlier for spring, growing, and fall seasons, respectively. The average onset of the snow cover season was 295 ± 10 DOY during the first 6 years of the study period (2013–2018), while during the last 2 years (2019–2020), it started about 15 days later, averaging at DOY 310 ± 6. Nevertheless, except for the fall season ($p_{t}$ = 0.008, $p_{w}$ = 0.037), these observed shifts were not statistically significant. Despite limited data coverage, the historical record confirms this pattern, particularly with the onset of the snow cover season occurring even earlier than in the initial years of the more recent dataset starting 2013.

**TABLE 1:** The start in day of year (DOY) and the length of each season over the study period (2013–2021) and the historical dataset (2002–2005).

Years	Start (DOY)				Length (day)			
	Snow cover	Spring	Growing	Fall	Snow cover	Spring	Growing	Fall
2002	275	128	185	234	218	—	49	41
2003	283	145	176	227	227	48	51	56
2004	—	—	172	234	—	27	62	—
2005	—	—	—	—	—	—	—	—
.	.	.	.	.	.	.	.	.
2013	305	—	—	—	210	—	—	—
2014	297	150	177	235	221	27	58	62
2015	279	153	174	229	225	21	55	50
2016	305	139	164	232	202	25	68	73
2017	287	141	170	228	231	29	58	59
2018	297	153	175	236	213	22	61	61
2019	305	145	167	222	197	22	55	83
2020	314	137	161	219	190	24	58	95
2021	—	138	160	220	—	22	60	85

#### 3.1.2 Vegetation Changes

**FIGURE 4:**
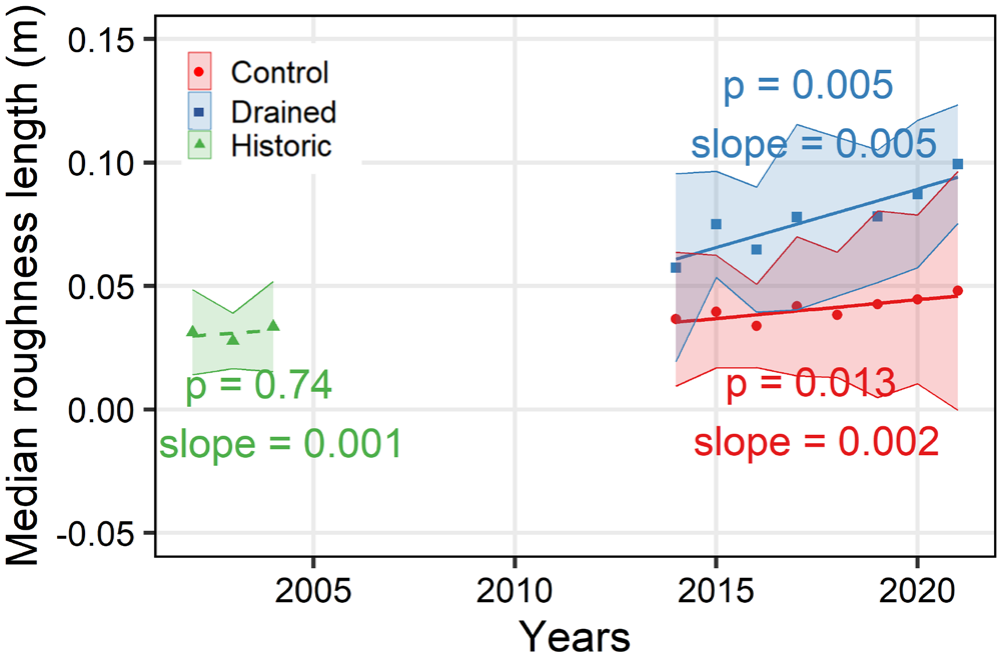
Median roughness length calculated over the control and drained site and the historic dataset. Here, the shaded areas represent the standard deviations. The linear trends for drained and control sites are represented by solid lines (significant p<0.05), while for the historic dataset, the trend is indicated by a dashed line (not significant p>0.05). Slope values of the linear trends are reported with a unit of m ${\text{year}}^{-1}$.

The vegetation community can be severely impacted by general warming trends in air temperatures and modified sub‐surface conditions caused by drainage, as reported by Kwon et al. (2016) for our site. As vegetation activity is one of the key drivers of carbon fluxes, any shift in vegetation community might have a strong impact on the overall carbon budget of the ecosystem. Since long‐term, consistent direct observations of vegetation communities are not available for our site, we used the aerodynamic roughness length ($z_{0}$) derived from eddy‐covariance tower observations as a proxy to track changes in vegetation structure over time (see Figure 4). To avoid excessive variations in $z_{0}$ in our calculations, we applied hard thresholds to the input parameters ($0.05<u_{*}<1.0$ and U>2.0), and restricted the atmospheric stability to near neutral boundary layer conditions ($-0.02<\frac{z}{L}$ < 0.02, where *L* is the Obukhov length). Compared to the historical dataset, which had a median $z_{0}$ of 0.032±0.020 m (year 2005 was omitted due to drainage), median $z_{0}$ values for the 2014–2021 observation period were found to be higher, with ($z_{0}=0.040\pm 0.032$ m) at the control and ($z_{0}=0.076\pm 0.032$ m) at the drained site. This suggests that, compared to historical records, the vegetation became taller and/or denser over both study sites. However, the more pronounced increase at the drained site suggests that the changes in vegetation structure were influenced by the long‐term disturbance from drainage. Moreover, the continued increase in roughness length differences between drained and control sites suggests that the vegetation is still adapting to changing environmental conditions. The evidence towards substantial shifts in vegetation conditions following drainage is further supported by a greenness index trend analysis in Landsat imagery (Nitze and Grosse 2016; Nitze et al. 2024) during July and August over the period 2003 to 2022 (see Figure S5). This plot clearly demonstrates that the vegetation around the drained site, specifically close to the drainage ditch, has become much greener over this observation period, while the wetter fetch area of the control site displays a rather neutral trend. Further support for these findings with additional ecosystem variables will be discussed in the next sections.

In summary, our data indicates that the combination of a later onset of snow cover and earlier snow melt has led to a shortening of the snow cover period. At the same time, the tendency towards an earlier start of the vegetation period did not lead to an extension of the growing season, since start and end dates of that season followed about the same trajectory over the years. In addition, we detected significant changes in the vegetation structure by observing the changes in $z_{0}$ over the time period. Compared to the historical data, $z_{0}$ at the drained site was more than twice as high, indicating notable changes towards taller and/or denser vegetation 15 years after drainage.

### 3.2 Annual ${\mathrm{CO}}_{2}$ and ${\mathrm{CH}}_{4}$ Budgets

**FIGURE 5:**
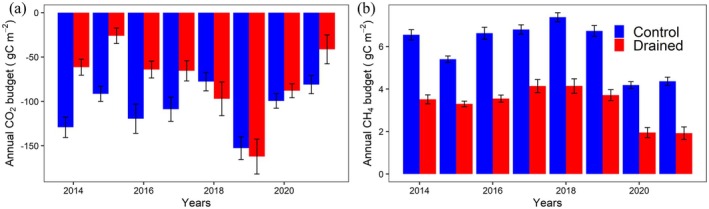
Net annual budget of (a) ${\mathrm{CO}}_{2}$, and (b) ${\mathrm{CH}}_{4}$ of drained (red), and control sites (blue). Uncertainties are represented with vertical lines for each year.

Both drained and control sites were found to be net sinks for ${\mathrm{CO}}_{2}$ between 2014 and 2021 (see Figure 5a). The long‐term average of annual cumulative ${\mathrm{CO}}_{2}$ fluxes was −75.7±41.7 and −107.5±25.5 gC m^−2^ for the drained and control sites, respectively, with differences in budgets between sites not being statistically significant ($p_{t}$ = 0.142, $p_{w}$ = 0.049). Over the study period, the control site had stronger ${\mathrm{CO}}_{2}$ uptake compared to the drained site in all data years, except for 2018 and 2019. For the control site, the observed interannual variability was relatively low, with the minimum annual uptake not smaller than −77.7 gC m^−2^, and only 2019 standing out as a strong outlier with uptake around −150 gC m^−2^. In contrast, the drained site showed pronounced interannual variability, with annual ${\mathrm{CO}}_{2}$ uptake in 2015 and 2021 falling about 66% and 45% below the long‐term average, respectively, while 2019 experienced an exceptionally high uptake, around double of the long‐term mean value.

Average annual ${\mathrm{CH}}_{4}$ emissions were 3.28±0.88 and 6.01±1.20 gC m^−2^ for the drained and control sites, respectively (see Figure 5b), with differences between sites statistically significant ($p_{t}$ = 0.0006, $p_{w}$ = 0.0002). ${\mathrm{CH}}_{4}$ emissions at the wet control site were considerably impacted by reduced precipitation (see Figure 1a) and corresponding lower water levels during the growing season, specifically in the later years of the observation period. As a consequence, ${\mathrm{CH}}_{4}$ emissions in 2020 and 2021 almost equalled those observed at the drained site in the preceding years. The drained site also reacted to these drier conditions, but net changes in annual ${\mathrm{CH}}_{4}$ were less pronounced: the average reduction at the control site was $2.31\pm 0.29\;\;\mathrm{gC}\;m^{-2}$ while at the drained site, it was $1.79\pm 0.37\;\mathrm{gC}\;\;m^{-2}$.

### 3.3 Seasonal ${\mathrm{CO}}_{2}$ and ${\mathrm{CH}}_{4}$ Budgets

**FIGURE 6:**
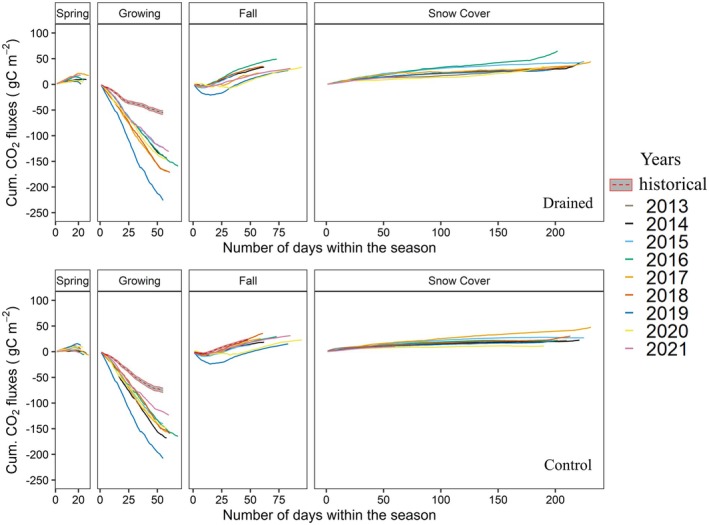
Cumulative sums of daily averaged ${\mathrm{CO}}_{2}$ fluxes separated by different seasons for drained site and control site between 2013 and 2021. ${\mathrm{CO}}_{2}$ fluxes from the historical dataset were represented as dashed lines (shaded region represents the standard error) as a reference only for the growing season in the drained site (2005) and growing and fall seasons for the control site (2002–2004). Because the length of each season differs in each year, the *x*‐axes across subplots have different ranges.

Figure 6 shows the cumulative ${\mathrm{CO}}_{2}$ fluxes for the drained and control sites across the four seasons defined in Section 3.1. Similar to the season length results, due to our seasonality definition, the snow cover seasons end in the following calendar year, for example, the cumulative sums for the 2014 snow cover season start in late 2014 and end in early 2015. Since our measurements started in July 2013, the snow cover season for 2013 was included in the cumulative plots, while other seasons in 2013 are omitted because either the start or end of these seasons depends on the maximum GPP, which cannot be specified for 2013. For the 2021 snow cover season, cumulative fluxes are plotted for only around 60 days (i.e., until the end of the calendar year). Additionally, as a reference, we included ${\mathrm{CO}}_{2}$ fluxes from the historical dataset 2002–05 (Kittler et al. 2016; Merbold et al. 2009; Corradi et al. 2005): Here we attributed data for the growing and fall seasons 2002–2004 to plots for the control site, and the growing season for 2005 was used to represent the drained site. Note that ${\mathrm{CO}}_{2}$ fluxes from the historical dataset were processed using a different software (TK3; Kittler et al. 2016), therefore comparisons are only made qualitatively throughout this manuscript. Compared to the historical data, for both control and drained sites results from the experiment phase starting 2013 more ${\mathrm{CO}}_{2}$ uptake is observed during the growing season, while fall season ${\mathrm{CO}}_{2}$ fluxes at the control site do not differ from the recent data. This increased uptake during the growing season may be attributed to the general warming trend over the Arctic, as this difference is also evident at the control site.

The period during which both sites act as a net carbon sink typically spans from late June to early September (data not shown). As shown in Figure 6, the majority of the annual carbon uptake and interannual variability in ${\mathrm{CO}}_{2}$ fluxes can be attributed to the relatively short growing season, with seasonal ${\mathrm{CO}}_{2}$ budgets ranging from −123.11 to −226.03 gC m^−2^ for the drained site and from −123.51 to −207.86 gC m^−2^ for the control site between 2014 and 2021. This stresses the importance of environmental conditions during the growing season on net annual ${\mathrm{CO}}_{2}$ budgets. Nevertheless, cold season emissions were found to be a significant carbon source, with net ${\mathrm{CO}}_{2}$ emissions during snow cover seasons between 2014 and 2021 corresponding to an average of 14.9%±4.4% and 12.4%±3.6% of the total annual respiration budgets for the drained and control site, respectively. In this context, the percentage contributions were found by adding up fluxes from snow cover periods within individual calendar years. Net ${\mathrm{CO}}_{2}$ emissions during the snow cover period for years 2013–2020 amounted to an average of 41.16 ± 11.01 (min: 28.99 gC m^−2^; max: 64.98 gC m^−2^) and 25.64 ± 10.54 gC m^−2^ (min: 11.16 gC m^−2^; max: 47.78 gC m^−2^) for the drained and the control sites, respectively. This difference in snow cover emissions between the two sites was found statistically significant ($p_{t}$ = 0.005, $p_{w}$ = 0.01). Our results emphasize that the ${\mathrm{CO}}_{2}$ fluxes during snow cover are not negligible, both in terms of total emissions and interannual variability, supporting the importance of year‐round observations in the Arctic permafrost region.

**FIGURE 7:**
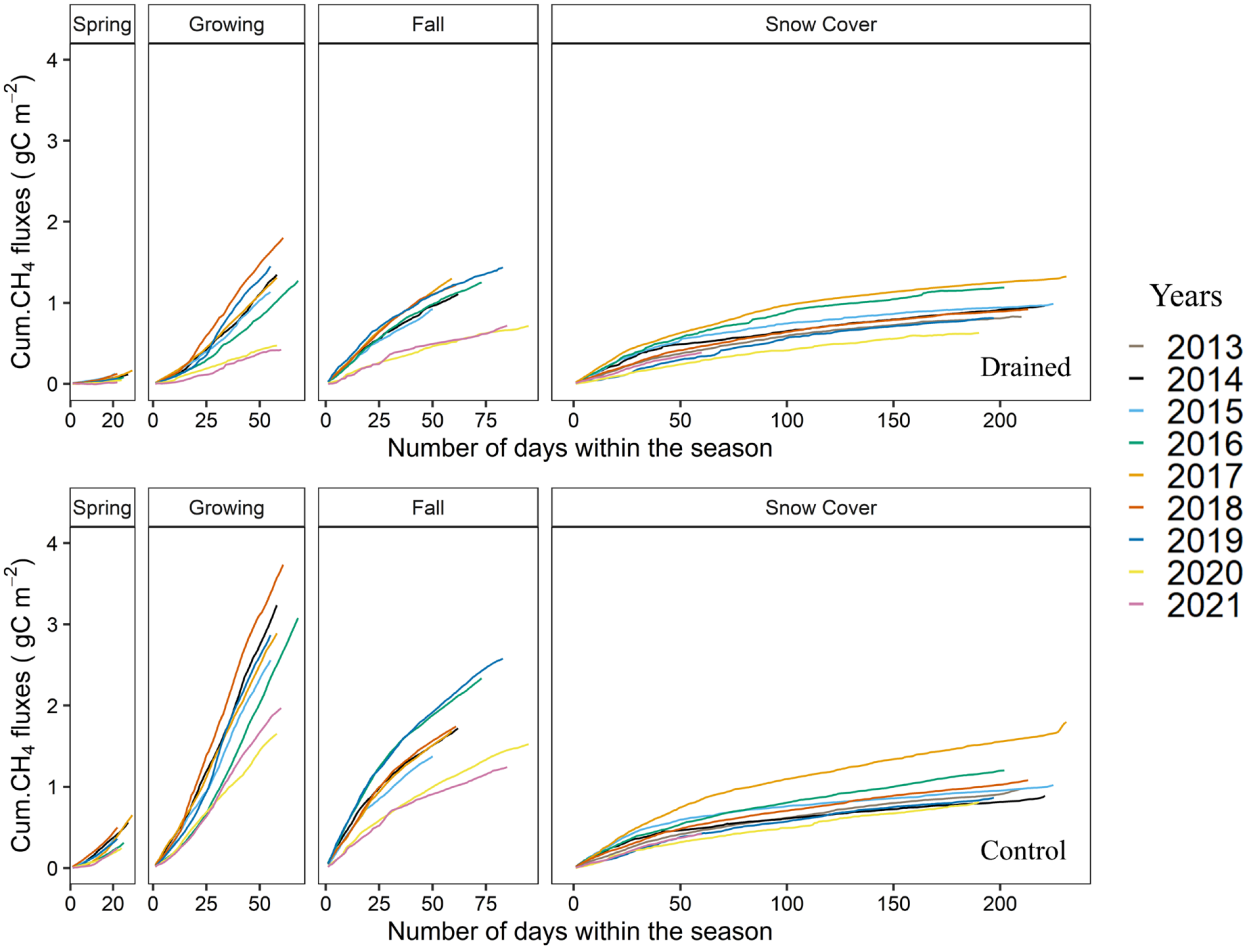
Cumulative sums of daily averaged of ${\mathrm{CH}}_{4}$ fluxes separated by different seasons for the drained site and the control site between 2013 and 2021. Because the length of each season differs in each year, the *x*‐axis of each subplot has different range, but all start from zero.

Cumulative ${\mathrm{CH}}_{4}$ fluxes broken up by season for the years 2013–2021 are illustrated for the drained and control sites in Figure 7. In this case, the highest interannual variability was found in the growing and fall seasons, while flux levels remained rather stable over the years during spring. The contributions of snow cover, fall, and growing seasons to the net annual ${\mathrm{CH}}_{4}$ budget are prominent for both sites, although with different rates. On average, drained site ${\mathrm{CH}}_{4}$ emissions during snow cover, fall, and growing seasons were found more or less equal (0.96±0.22, 1.08±0.27, and 1.15±0.48 gC m^−2^, respectively), while for the control site, the average ${\mathrm{CH}}_{4}$ emissions during the growing season (2.75±0.68 gC m^−2^) were found higher compared to fall (1.78±0.46 gC m^−2^) and snow cover (1.08±0.32 gC m^−2^) seasons. Furthermore, spring season ${\mathrm{CH}}_{4}$ emissions were relatively low (0.1±0.05 and 0.41±0.15 gC m^−2^ for the drained and control sites, respectively) compared to the other seasons, which is mainly due to relatively short spring season (on average about 24 days).

The average contributions of the snow cover season to the annual emissions were approximately 30.2%±6.7% and 18.1%±3.4% for the drained and control sites, respectively. These values are relatively high compared to findings reported in, for example, Kittler et al. (2017) and Rinne et al. (2007) (i.e., below 10%), whereas our results are comparable with observations reported by Rößger et al. (2022), who found ${\mathrm{CH}}_{4}$ contributions during the frozen season as high as 25% of the annual budget. Our year‐round observations between 2014 and 2021 show that relative flux contributions to the annual ${\mathrm{CH}}_{4}$ budget from outside the growing seasons were about 66.4%±7.4% and 54.6%±3.8% for the drained and control sites, highlighting the importance of non‐growing season measurements. High emission rates (> 50% of annual budgets) from non‐growing seasons reported in earlier studies were mostly attributed to the zero‐curtain period, which occurs when the insulation effects of snow cover delay the refreezing of the active layer in fall, even when air temperatures are far below zero (Zona et al. 2016; Rößger et al. 2022). Part of these emissions were observed as sporadic ${\mathrm{CH}}_{4}$ bursts during early freeze‐in (Zona et al. 2016; Mastepanov et al. 2008; Treat et al. 2018), in which ${\mathrm{CH}}_{4}$ emission processes remain active. This is also reflected in our observations, where high ${\mathrm{CH}}_{4}$ emissions were observed during the fall season at both sites (33.5%±4.0%, and 29.8%±5.9% for the drained and control sites, see also Figure 7).

### 3.4 Environmental Drivers of Carbon Fluxes

#### 3.4.1 Identifying Primary Drivers

**FIGURE 8:**
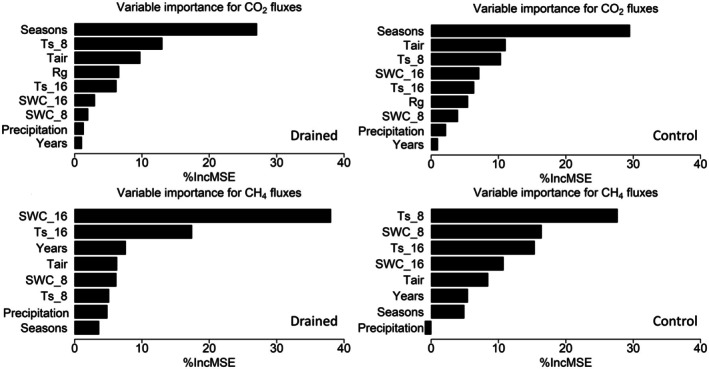
Relative importance of environmental controls for both monthly mean ${\mathrm{CO}}_{2}$ and ${\mathrm{CH}}_{4}$ fluxes at the drained and control sites. Here, Ts_8 and Ts_16 represent soil temperature at 8 and 16 cm depths, respectively, while SWC_8 and SWC_16 denote soil water content at 8 and 16 cm depths, respectively. The *x*‐axis shows the percentage increase in mean squared error, indicating that higher values correspond to higher importance in explaining the fluxes.

We analyzed the influence of environmental parameters on both interannual and interseasonal variations in ${\mathrm{CO}}_{2}$ and ${\mathrm{CH}}_{4}$ fluxes to identify underlying mechanisms and dominant control factors. The parameters considered include precipitation (Pr), air temperature ($T_{\mathrm{air}}$), soil temperatures at 8 cm ($T_{s-8}$) and 16 cm ($T_{s-16}$) below ground level, global radiation ($R_{g}$), and soil water content at 8 cm (${\mathrm{SWC}}_{8}$) and 16 cm (${\mathrm{SWC}}_{16}$) below ground level. Partial correlation analysis was conducted to identify the control variables that significantly impact carbon fluxes; Table 2 shows the partial correlation coefficients (τ) and their corresponding *p* values calculated by the Kendall method for monthly aggregated carbon fluxes and environmental controls. In addition, we used a random forest regressor to model the fluxes and specify the control variables with higher impact using the percentage increase in mean squared error from the random forest regressor (see Figure 8). In these driver analyses, all data including gap‐filled data were used. Although this might introduce some dependencies, we expect corresponding biases to be minimal due to the monthly aggregation, which helps to reduce the impact of any potential biases and smooths out anomalies introduced by the applied gap filling. From the partial correlation analyses, $R_{g}$ was the only variable that showed a significant correlation with ${\mathrm{CO}}_{2}$ fluxes for both sites. Although not found to be significant in this dataset, the relationship between ${\mathrm{CO}}_{2}$ fluxes and $T_{\mathrm{air}}$ and $T_{s-8}$ will be further investigated as temperature is known to influence respiration. As can be seen in the importance graphs in Figure 8 for ${\mathrm{CO}}_{2}$ flux models, the relationship with temperature can be confirmed, as $T_{\mathrm{air}}$ and both soil temperatures have relatively high importance at both sites, with $T_{s-8}$ chosen over $T_{s-16}$ for further investigation because of a higher importance. To represent the water level in both sites, ${\mathrm{SWC}}_{16}$ was included for further investigation, as its relative importance is higher than Pr and ${\mathrm{SWC}}_{8}$.

**TABLE 2:** Partial correlation coefficients of monthly mean environmental controls and carbon fluxes for both drained and control sites, along with their corresponding *p* values .

Controls	${\mathrm{CO}}_{2-d}$		${\mathrm{CO}}_{2-c}$		${\mathrm{CH}}_{4-d}$		${\mathrm{CH}}_{4-c}$	
	τ	*p*	τ	*p*	τ	*p*	τ	*p*
$T_{\mathrm{air}}$	−0.025	0.725	−0.094	0.186	−0.018	0.798	0.111	0.119
$T_{s-8}$	−0.046	0.516	−0.050	0.480	−0.056	0.434	0.142	0.046*
$T_{s-16}$	0.021	0.771	0.072	0.313	0.203	0.004*	0.116	0.101
$R_{g}$	−0.173	0.015*	−0.205	0.004*	—	—	—	—
Pr	−0.120	0.094	−0.074	0.297	0.358	0.000*	0.202	0.004*
${\mathrm{SWC}}_{16}$	0.050	0.488	0.083	0.247	0.199	0.005*	0.135	0.057
${\mathrm{SWC}}_{8}$	0.107	0.133	−0.012	0.863	0.06	0.429	0.156	0.028*

*Note:* The correlation coefficients and *p* values were calculated using the Kendall method for all flux values without separating by seasons, where * denotes statistically significant correlations.

Regarding ${\mathrm{CH}}_{4}$ fluxes at the drained site Pr, $T_{s-16}$, and ${\mathrm{SWC}}_{16}$ showed a significant correlation with ${\mathrm{CH}}_{4}$ fluxes, while at the control site, the correlation between ${\mathrm{CH}}_{4}$ fluxes and Pr, $T_{s-8}$, and ${\mathrm{SWC}}_{8}$ was statistically significant. The random forest model revealed a similar pattern (see Figure 8), with both soil temperatures indicating higher importance. This was expected, as WTD is lower at the drained site, the deeper soil variables become more relevant for ${\mathrm{CH}}_{4}$ fluxes. At the same time, Pr was found to be more important at the drained site compared to the control site, although its relative importance was not high especially at the control site. Since our partial correlation (see Table 2) showed the strongest relationship between Pr and ${\mathrm{CH}}_{4}$ fluxes at both sites, we decided to further investigate it. Therefore, for ${\mathrm{CH}}_{4}$ fluxes $T_{s-8}$, $T_{s-16}$, Pr, and ${\mathrm{SWC}}_{16}$ were selected for further investigation. As ${\mathrm{SWC}}_{16}$ measurements during snow cover season are not meaningful, these data were excluded from further analyses during that season.

Overall, seasons were found to be the most important feature for ${\mathrm{CO}}_{2}$ fluxes at both sites, while including years as a predictor did not improve the model performance. Also for ${\mathrm{CH}}_{4}$ fluxes, seasons were found to improve the model performance, although not as much as for ${\mathrm{CO}}_{2}$ fluxes. This underlines the importance of analyzing the impact of environmental controls and carbon fluxes based on seasonal variations.

#### 3.4.2 Influence of Drivers on Seasonal Carbon Fluxes

**FIGURE 9:**
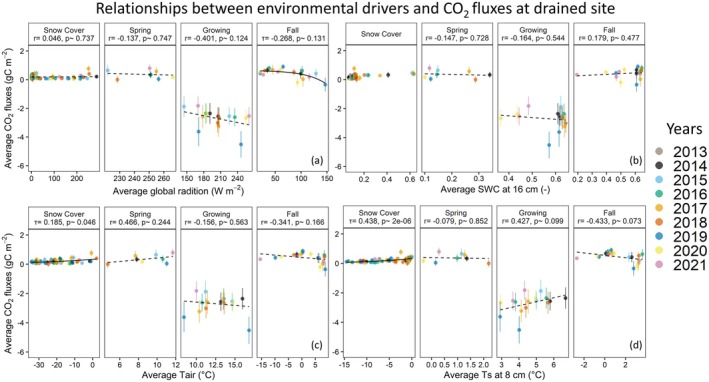
Relationships between ${\mathrm{CO}}_{2}$ fluxes and environmental variables global radiation (a), soil water content at 16 cm (b), air temperature (c), and soil temperature at 8 cm (d) for the drained site for each season. Each solid circle represents monthly averaged data, with uncertainties represented as vertical lines. The dashed lines are linear regression fits, while the solid lines represent non‐linear regressions fitted to the data. Correlation coefficients are provided as r for linear and τ for non‐linear regressions, along with the corresponding *p* values. Linear and non‐linear correlation coefficients were found via Pearson and Kendall methods, respectively.

After selecting the control variables for further investigation, we compared AIC parameters for linear and non‐linear regressions to identify the best models that explain carbon flux variations across different seasons (see Table S2). For temperature variables (i.e., $T_{\mathrm{air}}$, $T_{s-8}$, and $T_{s-16}$), we primarily used the Q10 function for non‐linear regressions, while for Pr, ${\mathrm{SWC}}_{16}$, and $R_{g}$ various non‐linear fittings were explored. All fitting functions that were used in this study are listed in Tables S3 and S4. For the drained site, the impact of the environmental controls on ${\mathrm{CO}}_{2}$ fluxes is illustrated in Figure 9. During snow cover and spring seasons, no clear trends were observed in ${\mathrm{CO}}_{2}$ fluxes across most environmental parameters, which can be mainly linked to prevailing very low fluxes in these seasons. However, as observed in previous studies (Webb et al. 2016; Natali et al. 2019), higher soil temperatures were generally associated with higher ${\mathrm{CO}}_{2}$ emissions during snow cover seasons, likely due to enhanced potential for microbial respiration under warmer temperatures (Natali et al. 2019). During the growing season, $R_{g}$ was primarily found to control the variability in ${\mathrm{CO}}_{2}$ fluxes, with $T_{\mathrm{air}}$ and ${\mathrm{SWC}}_{16}$ listed as a secondary control factors, while a slight negative correlation was observed between $T_{s-8}$ and the ${\mathrm{CO}}_{2}$ flux. In the fall season, $T_{s-8}$ became the major controlling parameter, followed by $R_{g}$ and $T_{\mathrm{air}}$. ${\mathrm{SWC}}_{16}$ seemed to become less dominant in influencing ${\mathrm{CO}}_{2}$ fluxes in the fall season. This may indicate that higher water levels alleviate drought stress compared to the growing season, while microbial decomposition may be slowed down under cooler soil conditions, with the potential for partial refreezing of the active layer.

**FIGURE 10:**
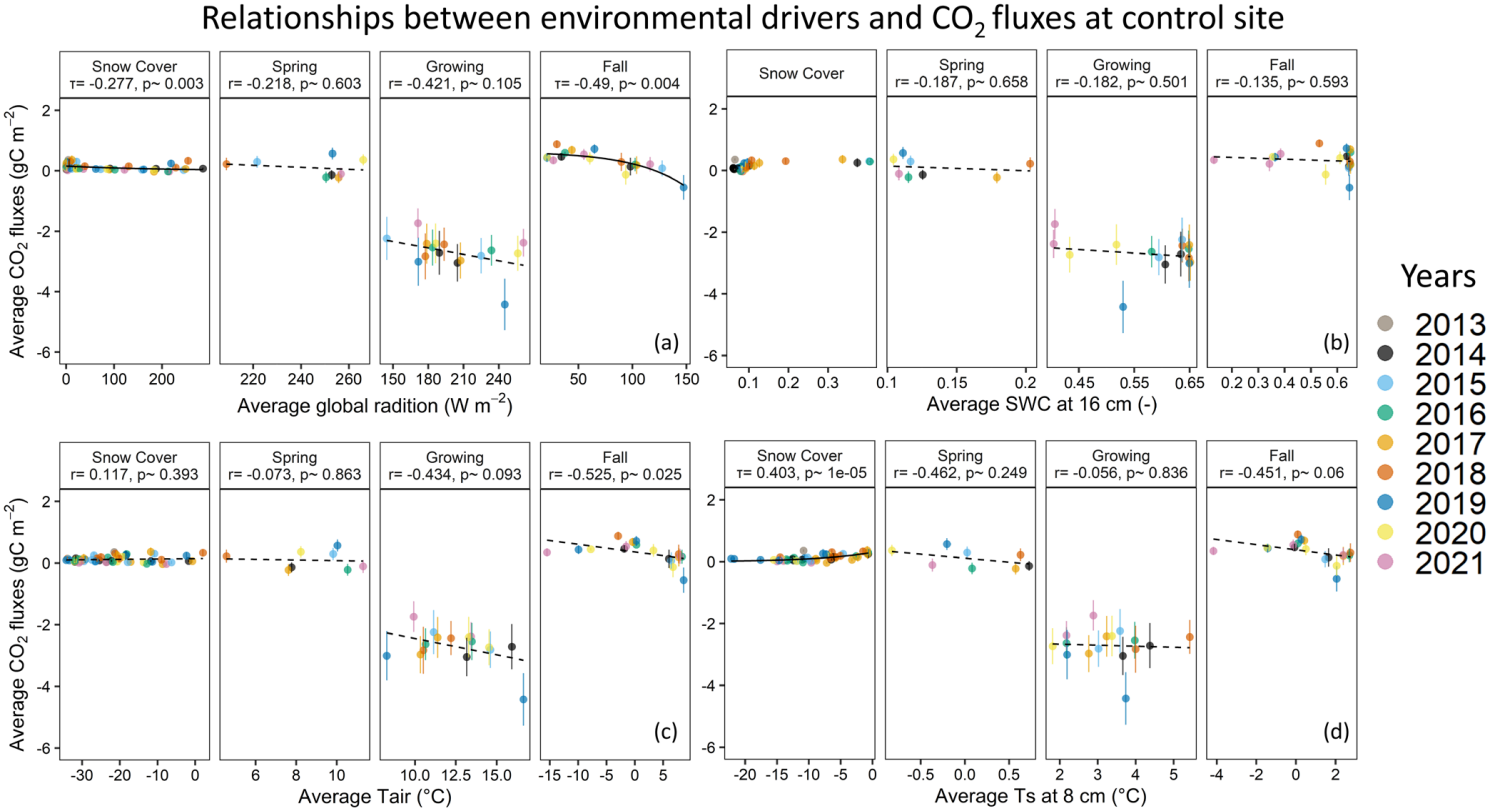
Relationship between ${\mathrm{CO}}_{2}$ fluxes and environmental variables global radiation (a), soil water content at 16 cm (b), air temperature (c), and soil temperature at 8 cm (d) for the control site for each season. Each solid circle represents monthly averaged data, with uncertainties represented as vertical lines. The dash lines are linear regression fits, while the solid lines represent non‐linear regressions fitted to the data. Correlation coefficients are provided as r for linear and τ for non‐linear regressions, along with the corresponding *p* values. Linear and non‐linear correlation coefficients were found via Pearson and Kendall methods, respectively.

For the control site, we generally observed similar trends to those found at the drained site (Figure 10), with exceptions in the relationships between ${\mathrm{CO}}_{2}$ fluxes and $T_{s-8}$ during the growing season. During growing and fall seasons, correlation coefficient between $T_{\mathrm{air}}$ and ${\mathrm{CO}}_{2}$ fluxes at the control site were higher than at the drained site. The slight negative correlation between $T_{s-8}$ and ${\mathrm{CO}}_{2}$ uptake at the drained site was mostly muted at the control site during the growing season. Furthermore, in contrast to the drained site, during fall season, among the environmental controls, $R_{g}$ became the major controlling parameter, followed by $T_{\mathrm{air}}$ and $T_{s-8}$. Overall, this suggests that aboveground conditions ($T_{\mathrm{air}}$ and $R_{g}$) are controlling the ${\mathrm{CO}}_{2}$ cycling during the growing season, while during snow cover seasons, belowground conditions ($T_{s-8}$) become more relevant for ${\mathrm{CO}}_{2}$ flux variability. This implies that the drainage impact on ${\mathrm{CO}}_{2}$ fluxes might be especially relevant during the cold season, as fluxes were about 1.6 times higher at the drained site compared to the control site.

**FIGURE 11:**
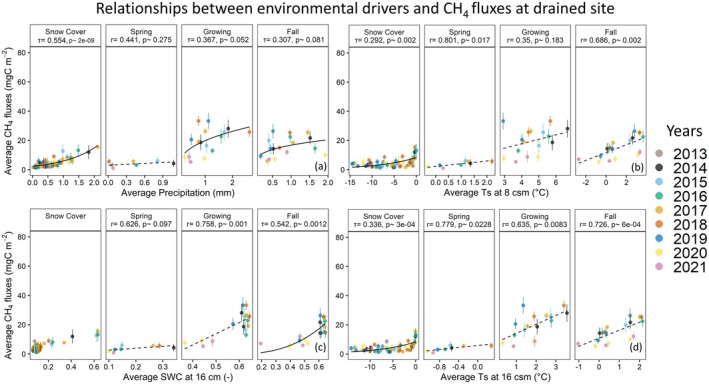
Relationship between ${\mathrm{CH}}_{4}$ fluxes and environmental variables average precipitation (a), soil water content at 16 cm (b), soil temperature at 8 cm (c), and soil temperature at 16 cm (d) for the drained site for each season. Each solid circle represents monthly averaged data, with uncertainties represented as vertical lines. The dash lines are linear regression fits, while the solid lines represent non‐linear regressions fitted to the data. Correlation coefficients are provided as r for linear and τ for non‐linear regressions, along with the corresponding *p* values. Linear and non‐linear correlation coefficients were found via Pearson and Kendall methods, respectively.

**FIGURE 12:**
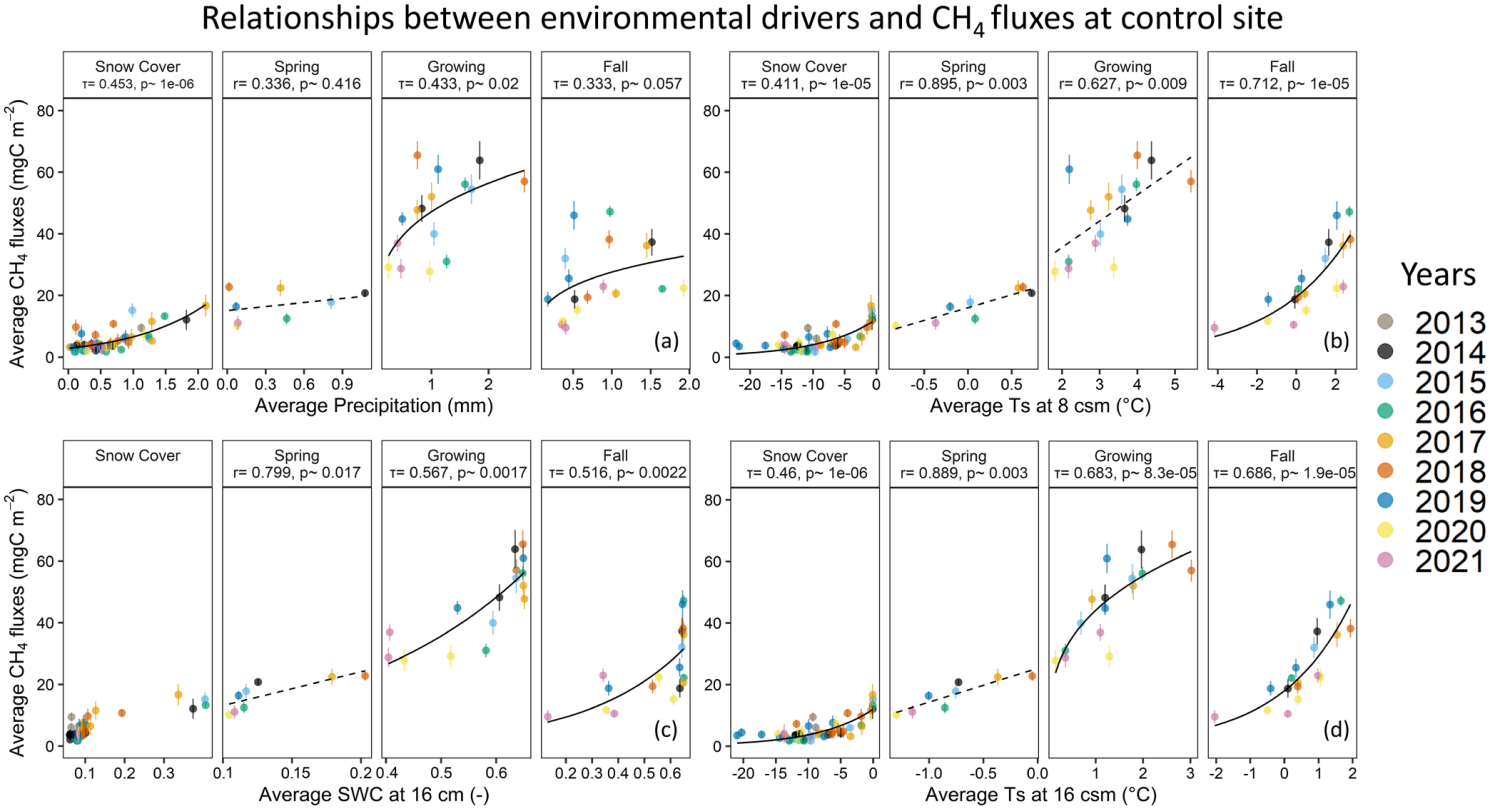
Relationship between ${\mathrm{CH}}_{4}$ fluxes and environmental variables average precipitation (a), soil water content at 16 cm (b), soil temperature at 8 cm (c), and soil temperature at 16 cm (d) for the control site for each season. Each solid circle represents monthly averaged data, with uncertainties represented as vertical lines. The dash lines are linear regression fits, while the solid lines represent non‐linear regressions fitted to the data. Correlation coefficients are provided as r for linear and τ for non‐linear regressions, along with the corresponding *p* values. Linear and non‐linear correlation coefficients were found via Pearson and Kendall methods, respectively.

At both sites, snow cover ${\mathrm{CH}}_{4}$ fluxes were primarily driven by $T_{s}$ and Pr (see Figures 11 and 12). The relatively mild (e.g., close to 0°C) monthly average soil temperatures during early and late snow cover season can be explained by the definition of the season, which is based exclusively on the albedo. This implies that the start of the snow cover season likely includes pockets of non‐frozen soils in the ground, which may influence the carbon cycle processes for an extended period of time, depending on the prevailing environmental conditions. Accordingly, in some years, the zero‐curtain period (Zona et al. 2016; Mastepanov et al. 2008; Treat et al. 2018) might be continuing far into the snow cover season, leading to high ${\mathrm{CH}}_{4}$ fluxes as shown in our results (see Figure 11b,d). During the spring season, across tested parameters, $T_{s}$ had the highest impact on ${\mathrm{CH}}_{4}$ emissions at both sites, while Pr did not show any strong influence. This might be related to the high water availability at both sites immediately after snow melt. At the control site, however, ${\mathrm{SWC}}_{16}$ can be listed as a secondary control parameter.

During the growing and fall seasons, ${\mathrm{CH}}_{4}$ emissions at both sites appeared to be mainly dependent on $T_{s-16}$. Although the correlation coefficients between ${\mathrm{CH}}_{4}$ fluxes and soil temperatures at 8 and 16 cm depths were similar, except growing season at the drained site, warming in the deeper soil had clearly higher impact on the absolute ${\mathrm{CH}}_{4}$ fluxes due the smaller temperature range in the deeper soil. We compared the slope values from the linear regressions for the drained site and found that the slope for $T_{s-16}$ was almost twice as high as that for $T_{s-8}$ during the fall seasons (5.5±1.3 and 2.71±0.72 gC $m^{-2}$ ${\mathrm{day}}^{-1}$°C^−1^, for $T_{s-16}$ and $T_{s-8}$, respectively) and the growing seasons (6.13±2.0 and 3.02±2.2 gC $m^{-2}$ ${\mathrm{day}}^{-1}$°C^−1^, for $T_{s-16}$ and $T_{s-8}$, respectively). This difference indicates that, as temperatures at deeper soil increase related to climate change or disturbance, ${\mathrm{CH}}_{4}$ fluxes might increase at a higher rate. Additionally, we observed some seasonal variations in ${\mathrm{CH}}_{4}$ flux dependencies, as both sites during the growing season showed a high correlation with Pr and ${\mathrm{SWC}}_{16}$, while in the fall season, the correlation between Pr and ${\mathrm{CH}}_{4}$ fluxes were mostly muted. Additionally, at the drained site, the influence of the ${\mathrm{SWC}}_{16}$ on growing season ${\mathrm{CH}}_{4}$ fluxes were higher compared to both $T_{s}$, while this was not the case at the control site.

### 3.5 Drainage Impact on Carbon Budgets and Influence of Environmental Controls

**FIGURE 13:**
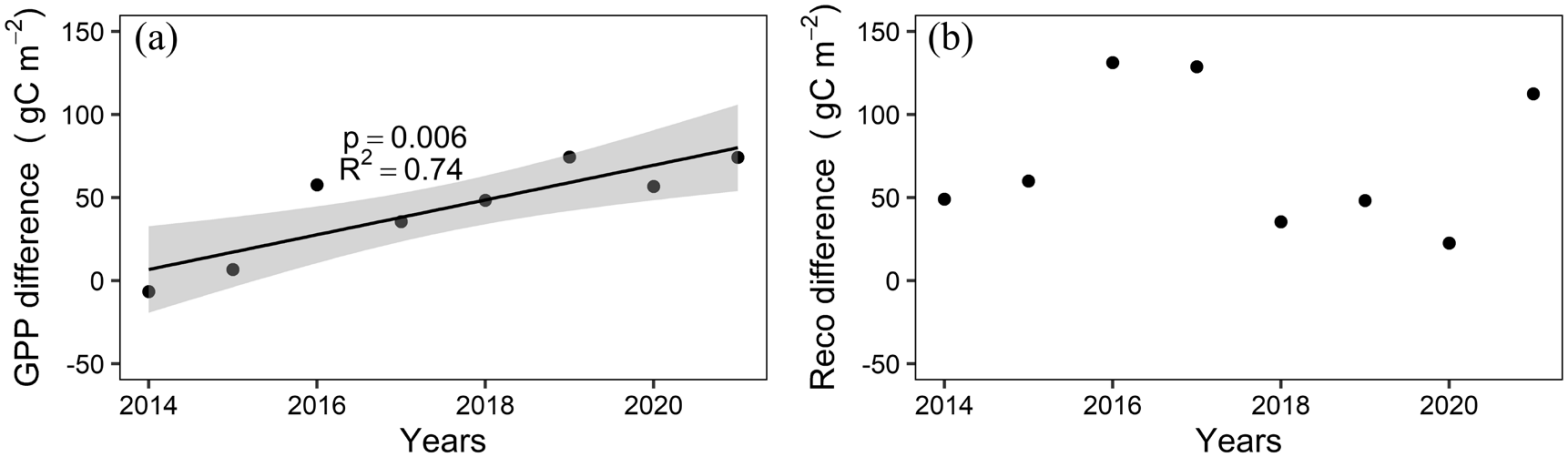
GPP (a) and Reco (b) difference between the drained and the control site (drained—control) over years. Each point represent annual GPP and Reco differences, and shaded area denotes the 95% of confidence interval. Here $R^{2}$ and *p* value were also provided.

The mean annual GPP budget was found to be 361.0±52.6, and 317.6±37.6 gC m^−2^ for the drained and control sites, respectively. Exceptionally high productivity was observed in 2019 at both sites, about 25% more than the annual average. The annual Reco budget was calculated as 277.8±29.4, and 204.4±29.6 gC m^−2^ for the drained and the control site, respectively, again with no significant longer‐term trend observed. The differences in total annual ${\mathrm{CO}}_{2}$ respiration budgets (2014–2021) between the drained and control sites were statistically significant ($p_{t}=0.0006$ and $p_{w}=0.001$), with constant higher respiration losses at the drained site compared to the control site. Such elevated ${\mathrm{CO}}_{2}$ emissions due to drained conditions were also reported by Kwon et al. (2016), who attributed this finding to changes in vegetation community structure and elevated soil temperatures in shallow layers. Indeed, the annual average soil temperatures at 8 cm depth were found as $-2.7^{{}^{\circ}}C\pm 1.6^{{}^{\circ}}C$, and $-4.8^{{}^{\circ}}C\pm 1.7^{{}^{\circ}}C$ for the drained and control sites, respectively, and this difference was found to be statistically significant ($p_{t}=0.024$ and $p_{w}=0.028$); that is, the drained site temperatures were always warmer. Over the study period, no clear trend was observed in Reco differences between the two towers (see Figure 13b).

Even though the long‐term average of ${\mathrm{CO}}_{2}$ fluxes showed a reduced sink strength as a consequence of the drainage disturbance (see also Section 3.2.1 above), contrary to the findings by Kwon et al. (2016), which covered a much shorter period of time, we did not observe a consistently reduced ${\mathrm{CO}}_{2}$ sink strength in annual ${\mathrm{CO}}_{2}$ budgets at the drained site (see also Figure 5a). Instead, in 2018 and 2019, the net sink strength for ${\mathrm{CO}}_{2}$ was found to be slightly higher at the drained site, albeit only by a very small amount. The fact that, compared to the control site, the drained site remained a higher net sink for ${\mathrm{CO}}_{2}$ in some more recent years indicates that the considerable increase in respiration rates is balanced by corresponding changes in differences between treatments for the photosynthetic uptake (GPP) over the same period. Indeed, a significant increase in GPP differences between the two sites with a coefficient of determination (*R* ^2^) of 0.74 (p=0.006) was observed over the years (Figure 13a). These trends indicate that consistently drier conditions continue to change the ecosystem even more than 15 years after the initial disturbance. The observed increase in GPP differences between these two sites might be partly related to the shift towards taller/denser vegetation cover, as suggested by the observed increase in $z_{0}$. This ongoing adaptation process is reinforced by different responses to shifts in climate forcing, which in this case intensifies the carbon turnover with higher fluxes in both GPP and respiration (see Figures S2 and S3).

In case of ${\mathrm{CH}}_{4}$ fluxes, about two times higher ${\mathrm{CH}}_{4}$ emissions were observed at the control site (see Figure 5b). Furthermore, the interannual variation at the control site was observed to be higher compared to the drained site. ${\mathrm{CH}}_{4}$ fluxes at both sites were highly affected by the dry conditions that occurred in the last 2 years of the study period (2020 and 2021), on average about 55% and 38% lower than annual average for the drained and control site, respectively. At the drained site, ${\mathrm{CH}}_{4}$ emissions during snow cover season had a higher contribution to the annual budget compared to the control site due to the net budget being relatively small. Nevertheless, our observations show that the average absolute ${\mathrm{CH}}_{4}$ emissions during the snow cover season were almost similar for the drained (0.95±0.22 gC m^−2^) and for the control sites (1.08±0.28 gC m^−2^). Assuming these sites showed no significant differences in ${\mathrm{CH}}_{4}$ flux signatures prior to the drainage installation, this finding suggests that artificially lowering the water table does not affect the ${\mathrm{CH}}_{4}$ budget during the cold season, but substantially lowers the ${\mathrm{CH}}_{4}$ emissions during other seasons. Although the net ${\mathrm{CH}}_{4}$ emissions are relatively small in the spring season, on average about four times higher ${\mathrm{CH}}_{4}$ emissions were observed at the control site compared to the drained site. During the growing season, control site ${\mathrm{CH}}_{4}$ emissions were about 2.5 times higher than those at the drained site. During the fall season, however, the differences in net ${\mathrm{CH}}_{4}$ emission levels were already reduced compared to the growing season, with fluxes about 63% higher ${\mathrm{CH}}_{4}$ at the control site (see Figure S4).

In addition to causing shifts in the annual and inter‐seasonal carbon flux budgets, the drainage was found to impact the ecosystem response to environmental controls. During the growing season, the ${\mathrm{CO}}_{2}$ uptake was slightly more sensitive to the $R_{g}$ at the drained site (slope=−7.94 per gC kW^−1^, p=0.12) compared to the control site (slope=−7.07 per gC kW^−1^, p=0.11). On the other hand, a relatively higher impact of air temperature to the ${\mathrm{CO}}_{2}$ uptake was observed at the control site (slope=−0.11 gC m^−2^/°C, p=0.09) compared to the drained site (slope=−0.05 gC m^−2^/°C, p=0.56). Differences were even more distinct for the ${\mathrm{CH}}_{4}$ emissions, for which during the growing season the impact of the $T_{s-16}$ was found to be much higher at the control site (slope=12.93 mgC m^−2^/°C, p=0.0002) compared to the drained site (slope=6.13 mgC m^−2^/°C, p=0.008). Similar to $T_{s-16}$, the control site ${\mathrm{CH}}_{4}$ emissions were more sensitive to changes in $T_{s-8}$ compared to the drained site. However, according to the partial correlation analyses of the ${\mathrm{CH}}_{4}$ fluxes at both sites, the dependency on Pr at the drained site was higher compared to the control site, where Kendall's τ between Pr and ${\mathrm{CH}}_{4}$ fluxes was 0.358 at the drained site and 0.202 at the control site. All fitting functions used in this section are provided in Tables S3 and S4.

## 4 Conclusions

In this study, we present observations from 8 years of data from two eddy‐covariance towers situated within the Arctic permafrost region. The unique disturbance experiment setup of our observation site (i.e., drained vs. control treatments) allowed us to observe and compare the impact of a consistently lowered water table depth manipulated by artificial drainage. As a second defining feature, the eddy‐covariance flux observations described and analyzed herein are continuous year‐round, meaning they include cold season flux data over the entire study period, allowing us to produce annual flux budgets and seasonal contributions to it.

Both sites were found to be net ${\mathrm{CO}}_{2}$ sinks and minor ${\mathrm{CH}}_{4}$ sources throughout the study period. Due to high interannual variability, we did not observe any clear long‐term trends in net carbon budgets at both sites. While drainage disturbance was found to only slightly reduce the ecosystem sink capacity for ${\mathrm{CO}}_{2}$, the impact of the lowered water table was significant on ${\mathrm{CH}}_{4}$ emissions, with annual ${\mathrm{CH}}_{4}$ emissions at the control site found to be almost two times higher than those observed at the drained site. At the same time, drainage intensified the carbon turnover rates, and higher budgets for both Reco and GPP were observed at the drained site compared to the control site. However, while there was no obvious trend in Reco, differences in GPP between these two treatments were found to be significantly increasing over the years, shifting from around balanced budgets in 2014 to annual GPP in the drainage area being more than 50 gC m^−2^ higher than in the control area in 2021. This finding indicates that even more than 15 years after the initial disturbance the site is not yet in equilibrium. This effect may also be responsible for a smaller reduction in net ${\mathrm{CO}}_{2}$ sink capacity following drainage reported herein, compared to an earlier assessment by Göckede et al. (2019) using fewer data years. One important factor in this context seems to be an ongoing trend towards taller and/or denser vegetation, as was shown by roughness length comparisons and LandSat imagery. These findings may imply that drained sites are more likely to turn into carbon sources with future climate change, since, contrary to respiration, further increases in GPP will eventually be limited by growing season length even under further warming. However, while the observed increases in vegetation height and density within the drainage‐affected areas continue, further increases in GPP can be expected, balancing enhanced respiration emissions from warmer soils. Accordingly, a conclusive evaluation on the drainage impact on the long‐term trajectory of carbon budgets under climate change can only be performed once the vegetation community becomes stable.

While our study confirmed that growing season fluxes dominate the annual budgets as well as the interannual variability, the non‐growing‐seasons had higher contributions than estimated in previous carbon budget assessments for this site. Despite very cold temperatures, the winter season alone contributed about 15% of the annual respiration budget, and about 25% of the annual ${\mathrm{CH}}_{4}$ emissions, with strong interannual variability particularly for off‐season fluxes of Reco highlighting the importance of the year‐round observations in Arctic permafrost regions. Regarding environmental controls, net ${\mathrm{CH}}_{4}$ emissions at the drained site were found to be more dependent on precipitation compared to the control site. Although incoming shortwave radiation was found to be the primary control of the ${\mathrm{CO}}_{2}$ uptake at both sites, the sensitivity was slightly higher at the drained site compared to the control site. These shifts in the influence of environmental controls following drainage imply that this type of disturbance will systematically alter the response of the ecosystem to future climate variability, an effect that needs to be considered in new generations of process models projecting permafrost carbon feedback with climate change.

In summary, these observational findings from a long‐term permafrost disturbance experiment are important to provide an outlook on how the carbon cycle processes may change following a persistent drying of Arctic wetlands. Considering the complex interaction of primary and secondary disturbance impacts observed in the context of our experiment, as well as long‐term trends, improves our understanding on permafrost—carbon feedback with climate change, and thus contributes to reducing uncertainties in prognostic simulations. Owing to our year‐round observations, this outlook is not restricted to the growing season, but also includes observational insights into non‐growing seasons, which were found to be significant contributors to annual ${\mathrm{CH}}_{4}$ and ${\mathrm{CO}}_{2}$ budgets in these regions.

## Author Contributions

**Abdullah Bolek:** conceptualization, data curation, formal analysis, methodology, visualization, writing – original draft, writing – review and editing. **Mark Schlutow:** formal analysis, methodology, writing – review and editing. **Theresia Yazbeck:** data curation, methodology, visualization, writing – review and editing. **Nathalie Triches:** visualization, writing – review and editing. **Martin Heimann:** conceptualization, funding acquisition, supervision, writing – review and editing. **Mathias Göckede:** conceptualization, funding acquisition, supervision, writing – review and editing.

## Conflicts of Interest

The authors declare no conflicts of interest.

## Supporting information


Data S1.

## Data Availability

The data that supports the findings of this study will be made available through the European Fluxes Database Cluster (https://www.europe‐fluxdata.eu/), using the site codes RU‐Che for the drained site, and Ru‐Ch2 for the control site.
