# Supplementary material for: Impact of Long‐Term Drainage on Carbon Fluxes in the High‐Latitude Permafrost Region
Source: Glob Chang Biol. 2025 Jul 15;31(7):e70346. doi: 10.1111/gcb.70346 (PMC12261280; doi:10.1111/gcb.70346)
Supplement: Supplementary file 1 — Data S1. [file GCB-31-e70346-s001.zip › Suppplementary.pdf]

Time series of CH_4 fluxes measured at the two towers are shown in Fig. S1 (a) and (b) for drained and control sites, respectively. Here, red colors represent data from closed path (CP) analyzers, light grey color is for gap filled using annual mean course (AMC), and dark grey representes gapfilled values from the XGBoost machine learning model (XGB),

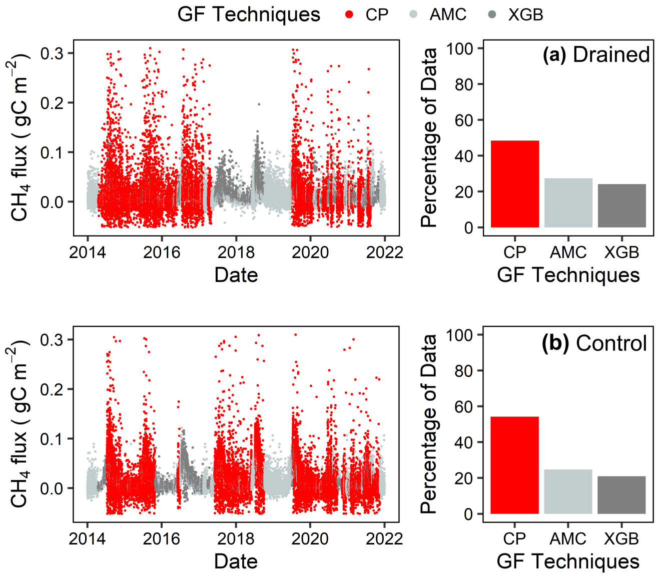

Figure S1. 30-min averaged CH_4 fluxes with different gap filling (GF) techniques represented with different colors for (a) Drained, and (b) Control Sites. Here, CP is data measured by the Closed Path instrument, AMC is gapfilled data using the annual mean course, and XGB is gapfilled data produced by the XGBoost machine learning model.

Seasonal flux budgets of both sites for all data years 2013-2021.

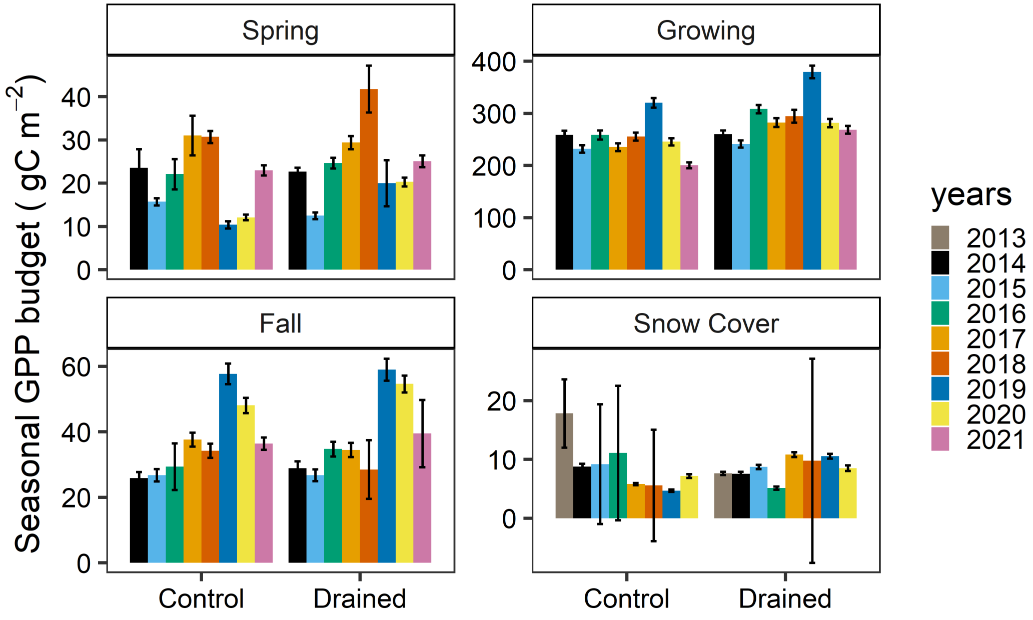

Figure S2. Net seasonal budget of Gross Primary Production (GPP) for all data years of both control and drained sites. The uncertainties are represented as vertical bars for each seasonal flux value.

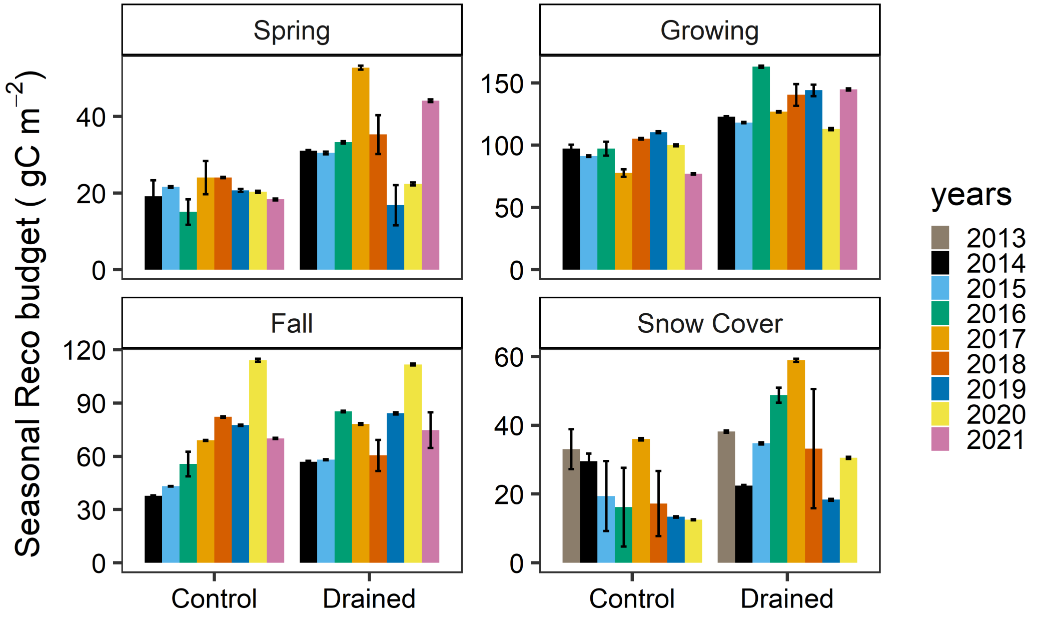

Figure S3. Net seasonal budget of ecosystem respiration (Reco) for all data years of both control and drained sites. The uncertainties are represented as vertical bars for each seasonal flux value.

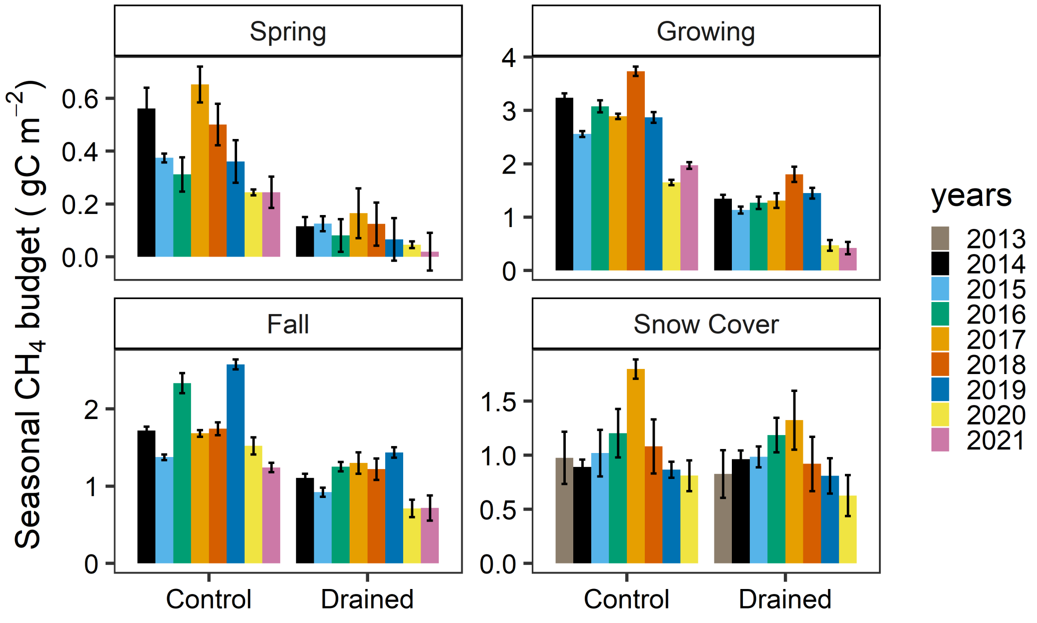

Figure S4. Net seasonal budget of CH_4 fluxes for all data years of both control and drained sites. The uncertainties are represented as vertical bars for each seasonal flux value.

Changes in greenness index captured from LandSat imagery between 2003 and 2022 during months July and August shown in Fig S5 (Nitze and Grosse 2016; Nitze et al., 2024).

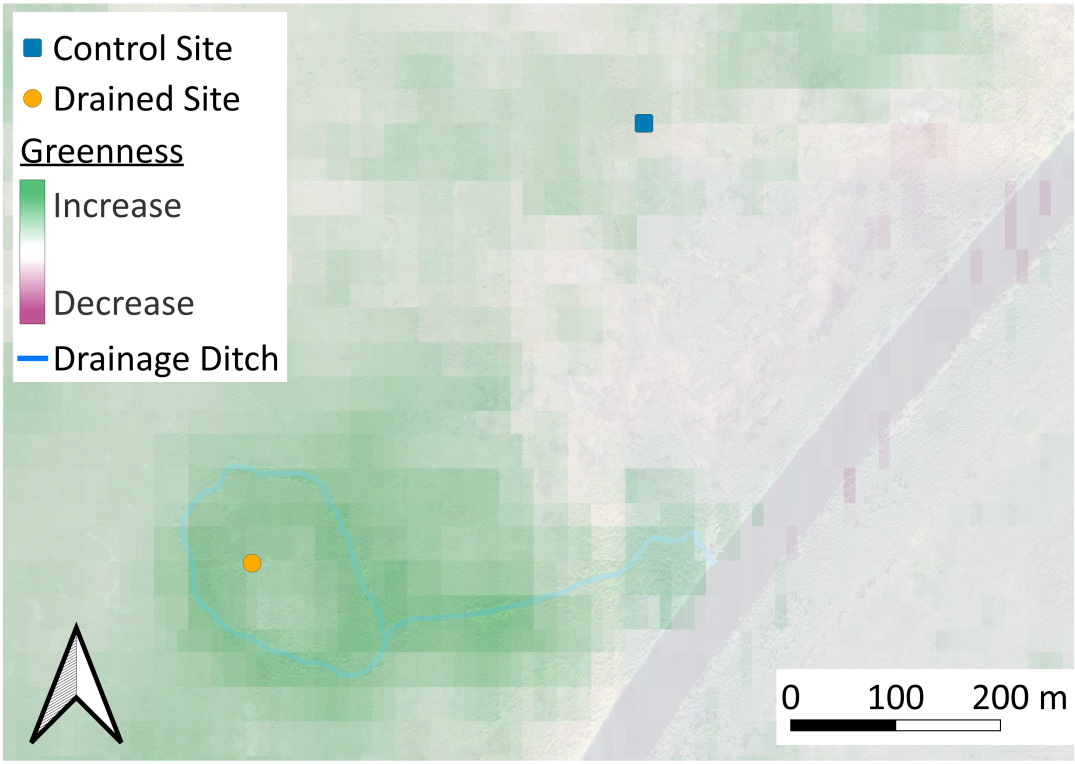

Figure S5. Spatially variable greenness trend from Landsat imagery between 2003 and 2022 during July and August. Green colors in this map indicate increased greenness while purple and white indicates decreased and no change over time, respectively. The drainage ditch was represented with blue color around the drained site represented by an orange solid circle, whereas the control tower was represented with a blue square.

Table S1. Details of the gap filling algorithms; XGB and AMC methods. Here, c and d subscripts denote as control and drained site.

Gap Filling Type	Gap Filled Variable	Predictors	RMSE
XGB	CO_{2_d}	CO_{2_c}, Tair, Rg, VPD, year, month, day, hour	1.12 $gCm^{-2}d^{-1}$
XGB	CO_{2_c}	CO_{2_d}, Tair, Rg, VPD, year, month, day, hour,	0.8 $gCm^{-2}d^{-1}$
XGB	CH_{4_d}	CH_{4_c}, T_{s-8_d},Tair, Rg, year, month, day, hour, T_{s-32_d}, $SWC16_d$	0.018 $gCm^{-2}d^{-1}$
XGB	CH_{4_c}	CH_{4_d}, T_{s-8_c},Tair, Rg, year, month, day, hour, T_{s-32_c}, $SWC16_c$	0.015 $gCm^{-2}d^{-1}$
XGB	T_{s-8_d}	T_{s-8_c}, Tair, Rg, VPD, year	0.71 °C
XGB	T_{s-8_c}	T_{s-8_d}, Tair, Rg, VPD, year	0.71 °C
XGB	T_{s-16_d}	T_{s-16_c}, Tair, Rg, VPD, year	0.61 °C
XGB	T_{s-16_c}	T_{s-16_d}, Tair, Rg, VPD, year	0.53 °C
XGB	T_{s-32_d}	T_{s-32_c}, Tair, Rg, VPD, year	0.54 °C
XGB	T_{s-32_c}	T_{s-32_d}, Tair, Rg, VPD, year	0.44 °C
XGB	SWC_{16-d}	SWC_c, Tair, Rg, VPD, year	0.10
XGB	SWC_{16-c}	SWC_d, Tair, Rg, VPD, year	0.29
AMC	CO_{2_d}	-	1.325 $gCm^{-2}d^{-1}$
AMC	CO_{2_c}	-	1.14 $gCm^{-2}d^{-1}$
AMC	CH_{4_d}	-	0.017 $gCm^{-2}d^{-1}$
AMC	CH_{4_c}	-	0.017 $gCm^{-2}d^{-1}$
AMC	T_{s-8_d}	-	2.697 °C
AMC	T_{s-8_c}	-	2.888 °C
AMC	T_{s-16_d}	-	2.899 °C
AMC	T_{s-16_c}	-	3.021 °C
AMC	T_{s-32_d}	-	3.346 °C
AMC	T_{s-32_c}	-	3.269 °C
AMC	SWC_{16-d}	-	0.09
AMC	SWC_{16-c}	-	0.09

Table S2. AIC values for the linear and non-linear regressions, here, first AIC values are for linear and second are for non-linear regressions.

	Drained				Control			
Regressions	Growing	Fall	Snow Cover	Spring	Growing	Fall	Snow Cover	Spring
$CO_2 \sim T_{air}$	36.4, 36.4	14.3, 15	-68.9, -69.9	5.8, 6.1	28.8, 28.3	10.6, 12.98	-91.5, -91.4	8.54, 8.54
$CO_2 \sim T_{s-8}$	33.6, 33.6	12.8, 13.9	-81.5, -83.3	7.7, 7.8	32, 32	12.3, 13.9	-116.5, -125.2	- , -
$CO_2 \sim R_g$	34.04, 34.2	8.5, 7.2	-59.4, -59.4	7.63, 7.65	29.02, 29.2	-0.5, -3.96	-97.1, -98.8	8.2, 8.03
$CO_2 \sim SWC_{16}$	36.4, 36.4	15.9, 16.1	-, -	7.6, 7.6	31.6, 31.4	16.1, 16.1	-, -	8.3, 8.2
$CH_4 \sim T_{s-8}$	118.5, 118.3	114.9, 115.1	270.7, 264.2	29.6, 30.7	124.3, 124.8	128.3, 124.7	268.1, 250.7	40.7, 40.4
$CH_4 \sim T_{s-16}$	112.4, 112.6	112.9, 112.6	267.5, 259.5	30.3, 32.2	115.8, 114.3	125.5, 120.5	261.9, 240.5	41.1, 42.9
$CH_4 \sim Prcp$	116.1, 115.0	124.2, 122.9	227.2, 223.4	36.0, 37.0	126.9, 126.0	142.3, 141.5	258.1, 252.3	52.6, 52.6
$CH_4 \sim SWC_{16}$	107.0, 107.1	113.8, 109.3	-, -	33.8, 34.4	116.1, 114.1	134.7, 133.6	-, -	45.4, 46.2

Table S3. Used fitting functions for environmental controls and CO_2 fluxes for both drained and control sites.

	Seasons	Equations: Drained	Equations: Control
$CO_2 \sim T_{air}$	Growing	$-2.14 - 0.045 * T_{air}$	$-1.383 - 0.106 * T_{air}$
	Fall	$0.465 - 0.016 * T_{air}$	$0.363 - 0.025 * T_{air}$
	Snow Cover	$0.448 * 1.33^{(T_{air}-10)/10}$	$0.147 + 0.001 * T_{air}$
	Spring	$-0.161 + 0.059 * T_{air}$	$0.185 - 0.01 * T_{air}$
$CO_2 \sim T_{s-8}$	Growing	$-3.942 + 0.268 * T_{s-8}$	$-2.598 - 0.034 * T_{s-8}$
	Fall	$0.55 - 0.081 * T_{s-8}$	$0.385 - 0.084 * T_{s-8}$
	Snow Cover	$0.92 * 2.749^{(T_{s-8}-10)/10}$	$0.966 * 3.185^{(T_{s-8}-10)/10}$
	Spring	$0.392 - 0.029 * T_{s-8}$	$0.115 - 0.258 * T_{s-8}$
$CO_2 \sim R_g$	Growing	$-1.085 - 0.008 * R_g$	$-1.28 - 0.007 * R_g$
	Fall	$-0.019 * e^{0.026 * R_g} + 0.647$	$-0.05 * e^{0.02 * R_g} + 0.643$
	Snow Cover	$0.186 + 0.00007 * R_g$	$0.156 * e^{-0.005 * R_g}$
	Spring	$1.027 - 0.003 * R_g$	$0.926 - 0.003 * R_g$
$CO_2 \sim SWC_{16}$	Growing	$-2.016 - 1.202 * SWC_{16}$	$-2.053 - 1.135 * SWC_{16}$
	Fall	$0.17 + 0.501 * SWC_{16}$	$0.487 - 0.289 * SWC_{16}$
	Snow Cover	—-	—-
	Spring	$0.46 - 0.46 * SWC_{16}$	$0.299 - 1.536 * SWC_{16}$

Table S4. Used fitting functions for environmental controls and CH_4 fluxes for both drained and control sites.

	Seasons	Equations	
		Drained	Control
$CH_4 \sim T_{s-8}$	Growing	$5.69 + 3.02 * T_{s-8}$	$18.52 + 8.551 * T_{s-8}$
	Fall	$11.7 + 2.71 * T_{s-8}$	$255.61 * 13.1^{(T_{s-8}-10)/10}$
	Snow Cover	$25.24 * 3.06^{(T_{s-8}-10)/10}$	$37.26 + 3.021^{(T_{s-8}-10)/10}$
	Spring	$2.162 + 1.889 * T_{s-8}$	$16.09 + 8.33 * T_{s-8}$
$CH_4 \sim T_{s-16}$	Growing	$8.06 + 6.13 * T_{s-16}$	$44.17 * {T_{s-16}}^{0.325}$
	Fall	$10.79 + 5.51 * T_{s-16}$	$2187.7 * 128.7^{(T_{s-16}-10)/10}$
	Snow Cover	$28.01 * 3.37^{(T_{s-16}-10)/10}$	$38.98 * 3.239^{(T_{s-16}-10)/10}$
	Spring	$5.84 + 3.4 * T_{s-16}$	$25.3 + 11.00 * T_{s-16}$
$CH_4 \sim \mathrm{Prcp}$	Growing	$19.888 * \mathrm{Prcp}^{0.398}$	$47.2 * \mathrm{Prcp}^{0.258}$
	Fall	$16.78 * \mathrm{Prcp}^{0.282}$	$27.59 * \mathrm{Prcp}^{0.263}$
	Snow Cover	$2.369 * e^{0.92 * \mathrm{Prcp}}$	$2.817 * e^{0.851 * \mathrm{Prcp}}$
	Spring	$3.042 + 2.1 * \mathrm{Prcp}$	$15.13 + 4.308 * \mathrm{Prcp}$
$CH_4 \sim SWC_{16}$	Growing	$-24.34 + 76.39 * SWC_{16}$	$7.915 + e^{3.013 SWC_{16}}$
	Fall	$1.05 * e^{4.633 * SWC_{16}}$	$5.499 * e^{2.684 * SWC_{16}}$
	Snow Cover	—-	—-
	Spring	$1.394 - 12.795 * SWC_{16}$	$2.18 + 109.6 * SWC_{16}$
